# Acceptability, equity, and feasibility of using antipsychotics in children and adolescents with autism spectrum disorder: a systematic review

**DOI:** 10.1186/s12888-020-02956-8

**Published:** 2020-11-25

**Authors:** Gian Loreto D’Alò, Franco De Crescenzo, Laura Amato, Fabio Cruciani, Marina Davoli, Francesca Fulceri, Silvia Minozzi, Zuzana Mitrova, Gian Paolo Morgano, Franco Nardocci, Rosella Saulle, Holger Jens Schünemann, Maria Luisa Scattoni, on behalf of the ISACA guideline working group, Raffaella Tancredi, Angelo Massagli, Giovanni Valeri, Corrado Cappa, Serafino Buono, Giuseppe Maurizio Arduino, Alessandro Zuddas, Laura Reali, Massimo Molteni, Claudia Felici, Concetta Cordò, Lorella Venturini, Cristina Bellosio, Emanuela Di Tommaso, Sandra Biasci, Clelia M. Duff, Simona Vecchi, Michele Basile

**Affiliations:** 1 Department of Epidemiology, Lazio Regional Health Service, Via Cristoforo Colombo, 112, 00154 Rome, Italy; 2 Department of Psychiatry, University of Oxford, Oxford, UK; 3 Pediatric University Hospital-Department (DPUO), Bambino Gesù Children’s Hospital, Rome, Italy; 4 Research Coordination and Support Service, Istituto Superiore di Sanità, Viale Regina Elena 299, 00161 Rome, Italy; 5 Department of Health Research Methods, Evidence and Impact (formerly Clinical Epidemiology and Biostatistics), McMaster GRADE Centre, McMaster University, Hamilton, ON Canada; 6 Department of Medicine, McMaster University, Hamilton, ON Canada

**Keywords:** Autism Spectrum disorder, Antipsychotic agents, Systematic review, Guideline, GRADE approach

## Abstract

**Background:**

It is unclear whether the administration of antipsychotics to children and adolescents with autism spectrum disorders (ASD) is acceptable, equitable, and feasible.

**Methods:**

We performed a systematic review to support a multidisciplinary panel in formulating a recommendation on antipsychotics, for the development of the Italian national guidelines for the management of ASD. A comprehensive search strategy was performed to find data related to intervention acceptability, health equity, and implementation feasibility. We used quantitative data from randomized controlled trials to perform a meta-analysis assessing the acceptability and tolerability of antipsychotics, and we estimated the certainty of the effect according to the GRADE approach. We extracted data from systematic reviews, primary studies, and grey literature, and we assessed the risk of bias and methodological quality of the published studies.

**Results:**

Antipsychotics were acceptable (dropouts due to any cause: RR 0.61, 95% CI 0.48–0.78, moderate certainty of evidence) and well tolerated (dropouts due to adverse events: RR 0.99, 95% CI 0.55–1.79, low certainty of evidence) by children and adolescents with ASD. Parents and clinicians did not raise significant issues concerning acceptability. We did not find studies reporting evidence of reduced equity for antipsychotics in disadvantaged subgroups of children and adolescents with ASD. Workloads, cost barriers, and inadequate monitoring of metabolic adverse events were indirect evidence of concerns for feasibility.

**Conclusion:**

Antipsychotics in children and adolescents with ASD were likely acceptable and possibly feasible. We did not find evidence of concern for equity.

**Supplementary Information:**

The online version contains supplementary material available at 10.1186/s12888-020-02956-8.

## Introduction

Autism spectrum disorder (ASD) is an early onset, neurodevelopmental disorder that causes a broad set of socio-communication deficits and restricted or repetitive behaviors. ASD is commonly associated with problem behaviors such as hyperactivity, irritability, and self-harm [1, 2]. ASD symptomatology causes reduced functioning, regardless of intellectual ability [3, 4]. The prevalence of ASD worldwide is about 1–2% [5], while in Italy, it is 1.14–1.3% [6, 7], with a male: female ratio of about 4:1. About 48% of children with ASD are affected by a form of intellectual disability [8, 9]. In the United Kingdom and the USA, the estimated lifelong costs to support an individual with ASD from the societal perspective range between 1.2 to 2 million euros, depending on the presence of intellectual disability [10].

Irritability and aggression are treatment targets for the use of antipsychotics in ASD [11]. The food and drug administration (FDA) approved risperidone and aripiprazole for the treatment of irritability in ASD, while there is no pharmacological treatment that has been proven to be effective in treating core symptoms [12].

It is not apparent whether antipsychotics are acceptable and feasible to a population of children and adolescents with ASD. Parents could be reluctant to administer antipsychotics to their children, and concern towards adverse events often leads parents to shift towards complementary and alternative medicines [12, 13]. Adverse events related to antipsychotics, such as increased appetite, weight gain, diabetes mellitus, and hyperlipidemia [14, 15], may also discourage clinicians from prescribing them in children and adolescents with ASD. Adverse events can as well lead to treatment discontinuation and switch to other drugs. Furthermore, children with ASD and developmental disabilities often show troubles in swallowing medications [16]. Parents often have to manage pill-swallowing difficulties on their own; they often use some form of coercion to achieve immediate compliance, but such behavior could lead children to develop anxiety, aversion to pills, and increased food selectivity [17].

In this study, we aimed to systematically review the evidence on acceptability, equity, and feasibility of antipsychotics in children and adolescents with ASD.

## Methods

### Context

The Italian National Institute of Health (in Italian: Istituto Superiore di Sanità – ISS) is currently developing evidence-based guidelines for the diagnosis and treatment of ASD in children and adolescents. Equity, acceptability, and feasibility are considered contextual factors able to influence recommendations developing when using the grading of recommendations assessment, development, and evaluation (GRADE) evidence to decision framework. We conducted this systematic review to support the ISS autism guidelines panel in formulating a recommendation on the use of antipsychotics, according to the ISS manual [18]. This project was carried out in conjunction with a multidisciplinary panel, which included subject experts, people with ASD, and their caregivers.

### The questions

We addressed the following clinically and public health relevant questions on the use of antipsychotics for children and adolescents with ASD:

- What would be the impact of antipsychotics on health equity?
- Are antipsychotics acceptable to key stakeholders?
- Are antipsychotics feasible to implement?

### Population

Children and adolescents aged 0–18 years, of both genders, with a primary diagnosis of autism spectrum disorder.

### Intervention

Antipsychotics selected by Guidelines panel members, including aripiprazole, clozapine, haloperidol, levosulpiride, lurasidone, olanzapine, risperidone and trifluoperazine.

### Outcomes

Equity: differences in the effectiveness of the intervention or in the importance of the problem predictable based on basic conditions present within disadvantaged subgroups [19, 20].

Acceptability: the probability for the key stakeholders to agree with the distribution of the net benefits, harms, and costs; the costs, or undesirable effects, to be paid in the short term to achieve the desirable effects or benefits in the future; the values associated with the desirable or undesirable effects [19]; discontinuation due to any cause when comparing the intervention versus placebo.

Feasibility: sustainability of the intervention considering barriers and facilitators to its implementation [19].

### Types of studies included

Inclusion and exclusion criteria were different for the purposes of quantitative and qualitative synthesis and were established a priori before conducting database searches.

### Quantitative synthesis

For the meta-analysis on acceptability, we included only RCTs. The inclusion of non-randomized studies in the meta-analysis would have introduced bias, as observational studies are prone to selection bias and confounding by indication. Studies eligible for inclusion: randomized controlled trials and non-randomized controlled trials (such as quasi-randomized, observational and experimental studies) evaluating acceptability, equity and feasibility of APs in children and adolescents with ASD; as we found a sufficient number of RCTs reporting data on the above-mentioned outcomes, we did not include non-randomized trials in the meta-analysis. We excluded: studies that that did not evaluate or report data about at least one criterion among acceptability, equity or feasibility; studies comparing two pharmacological interventions, without placebo arm; augmentation trials with no placebo arm; studies presenting pooled or post-hoc analyses of RCTs; studies whose design, population or intervention did not meet our inclusion criteria.

### Qualitative synthesis

All types of randomized and non-randomized studies (including systematic reviews, surveys, cohort, or cross-sectional studies) evaluating acceptability, equity and feasibility of APs in children and adolescents with ASD as a major part of the study. All types of studies that did not assess acceptability, equity and feasibility were excluded; studies whose population or intervention did not meet our inclusion criteria were also excluded.

### Literature search

The literature search was conducted up to January 2019. No language filters were applied. We searched Central, Medline, Embase, Web of Science, and PsycInfo. The search strategies are reported in Additional file 1**.**

### Study selection and data extraction

Two reviewers (GLD, FDC) independently screened titles and abstracts of the publications obtained by the search strategies. The same authors independently assessed the inclusion of the full text of studies that potentially satisfied inclusion criteria. Disagreements were resolved by a consensus meeting or by a third reviewer (LA).

Two reviewers (GLD, FDC) independently extracted data. The full methodology followed for extracting data for each considered domain is in Additional file 2 [19, 21, 22].

### Data analysis and synthesis

Quantitative data extracted from RCTs (discontinuation due to any cause and discontinuation due to adverse event) were analyzed by the Risk Ratio (RR) using a random effect model [23] and expressing uncertainty with its 95% confidence interval (CI). Heterogeneity between studies has been investigated by the Q-test, by the I-squared statistic (I-squared equal to or more than 50% was considered indicative of heterogeneity), and by visual inspection of the forest plots. To investigate differences in pooled effect estimates related to type of antipsychotic drug, we conducted a subgroup analysis presenting sub-total effect estimates for each drug in the forest plots. In order to evaluate the presence of a possible publication bias, we used the funnel plot method which shows on the x-axis the magnitude of the effect (effect size) versus the estimated precision of the study (most commonly the standard error of the estimated association) on the y-axis. In the absence of publication bias, the points representing the studies have a roughly symmetric funnel shape, in contrast, when there is publication bias, smaller, less precise studies show a significant positive effect suggesting that small negative studies were not published and leading to an asymmetric funnel.

For the two outcomes, we produced a summary of findings table as advised by the Grading of Recommendations Assessment, Development and Evaluation (GRADE) Working Group [19, 24–27]. After evaluating the study limitations, indirectness, inconsistency, imprecision of effect estimates, and risk of publication bias, we attributed four levels (high, moderate, low, very low) to the certainty in the evidence, accompanying the results with a narrative statement [28].

We synthetized narratively data retrieved from systematic reviews and observational studies on acceptability, feasibility, and equity of antipsychotics in children and adolescents with ASD.

We used the GRADE Evidence to Decision (EtD) framework criteria to present and summarize the results.

### Quality assessment

Depending on the type of studies included we used different methods to assess the methodological quality. Regarding the studies included in the quantitative synthesis (RCTs), we used the Cochrane tool for risk of bias assessment [29], evaluating: sequence generation; allocation concealment; blinding of participants, providers and outcome assessors; incomplete outcome data; selective reporting. We then created a ‘Risk of bias’ table for the included studies, thus indicating the study’s performance (low, high, or unclear risk of bias) in each of the domains mentioned above. Regarding the studies included in the qualitative synthesis we used the Newcastle Ottawa Scale (NOS) for the quality assessment of case-control and cohort studies [30], and a modified version of NOS [31] for cross-sectional studies. We planned to use, but did not, the Risk Of Bias In Non-randomized Studies of Interventions (ROBINS-I) tool, as we did not find any non-randomized study comparing APs to placebo in our population reporting outcomes of interest. To assess the methodological quality of the systematic reviews and meta-analyses included in our study, we used Amstar 2 [32].

## Main text

### Search strategy results

**Fig. 1:**
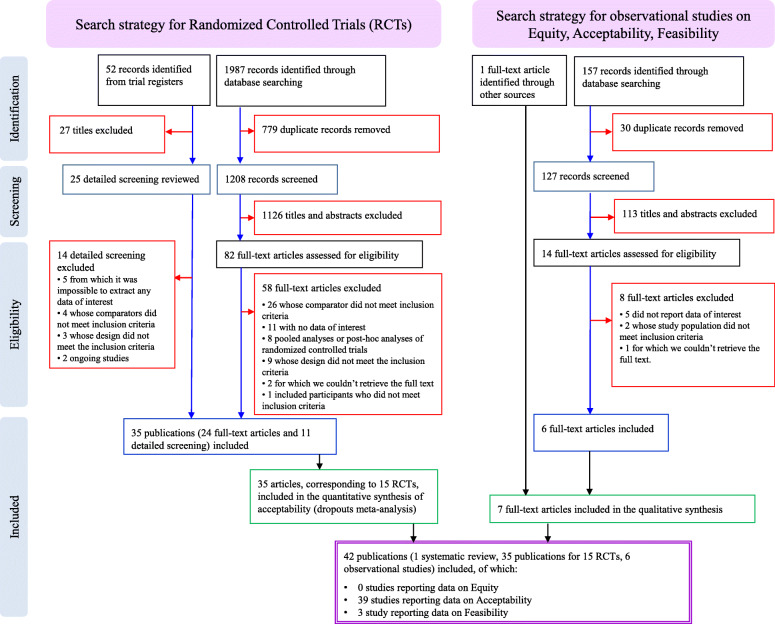
Flow chart

Through bibliographic searches, we identified 1388 reports after removing duplicates; we excluded 1266 studies on the basis of title and abstract. We retrieved 122 articles in full text for more detailed evaluation, 80 of which we excluded after reading the full text; of the remaining 42 studies, 35 were primary or secondary references referring to 15 RCTs [33–47], 6 were observational studies [48–53], and 1 was a systematic review [54]. See Fig. 1 for the flow chart, and Additional file 3 for the full references for included and excluded studies. We reported the methodological quality of included studies in Additional files 4, 5 and 6.

### Equity

We did not find any study that reported data concerning equity in the use of antipsychotics in children and adolescents with ASD.

### Acceptability

#### Acceptability by parents

Bowker et al. (2011) [48] carried out an online survey to investigate the motivations behind the therapeutic choice of parents of children and adolescents with ASD and their perception of the changes following the therapy undertaken. Nine hundred seventy questionnaires were collected (93% from North America), 96.4% from children and adolescents with ASD, and completed by parents. The survey showed that 77% of subjects had received therapy for ASD during their lifetime, but only 14.6% were on medication at the time the study was conducted. According to the parent’s judgment of the effectiveness of the treatment, the area of functioning that benefited most from drug therapy was behavioral (31.9%).

In contrast, a smaller number of parents indicated more notable benefits in the cognitive (16.3%), attention (14.2%), linguistic (12.8%), social (11.3%), and physical (3.5%) areas. Drug therapy was discontinued by 20% of the population. The reasons for discontinuation were mainly lack of efficacy (43% in total and 17% among those who had used antipsychotics at least once) and adverse events (29% in total and 11% among those who had used drug therapy at least once).

The same study reported some factors that parents considered determinant in choosing the type of treatment for their children with ASD, including:

- opinions about the causes of the disorder;
- parental style;
- lifestyle;
- socio-economic status;
- ease of access to services and care;
- impact of the media and the testimonies of other families.

The study concluded that the scientific evidence is not, for parents, decisive for the choice of treatments. Therapies supported by evidence are often underused, while frequently, non-evidence-based treatments are used. Non-evidence-based treatments can be potentially harmful, and the scientific community has a responsibility to explore all possible avenues to help parents to make well-informed decisions.

A survey [50] (*n* = 96 questionnaires administered), performed in the context of an RCT on the administration of risperidone versus placebo, found the following significant correlations between parent satisfaction and demographic characteristics: a) low income to poor satisfaction with the number of visits (*p* = 0.003); b) the child’s IQ ≥45 and white ethnicity with poor satisfaction to the learning tests (*p* = 0.043); c) the lower education to poor satisfaction with the behavioral assessment (*P* = 0.033).

#### Acceptability by children and adolescent with ASD

**Fig. 2:**
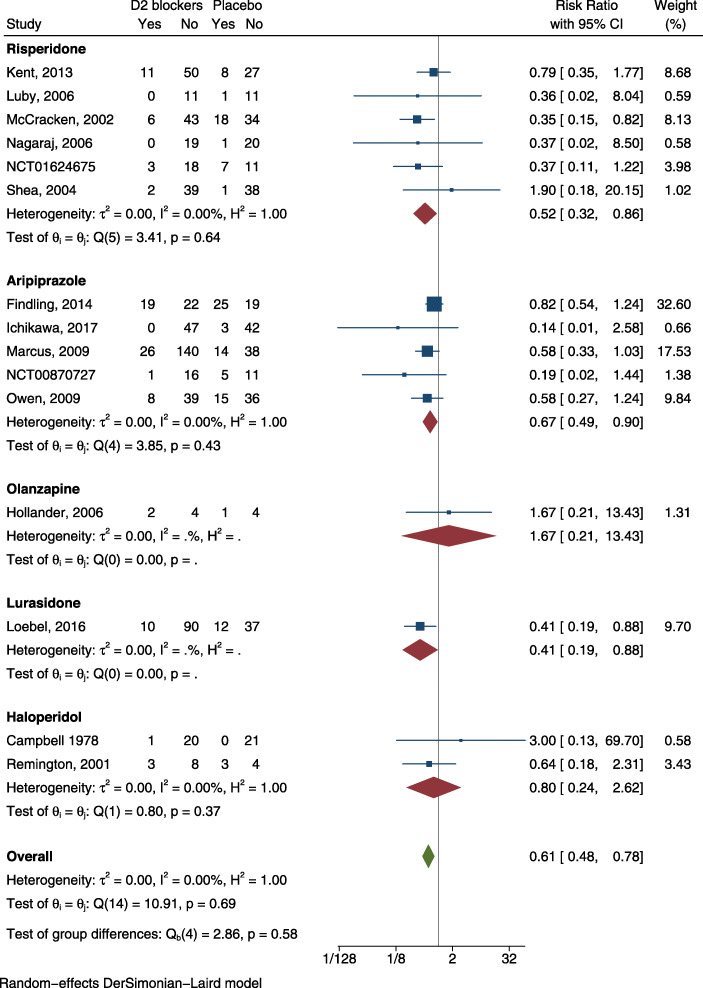
Forest plots for discontinuation due to any cause

**Fig. 3:**
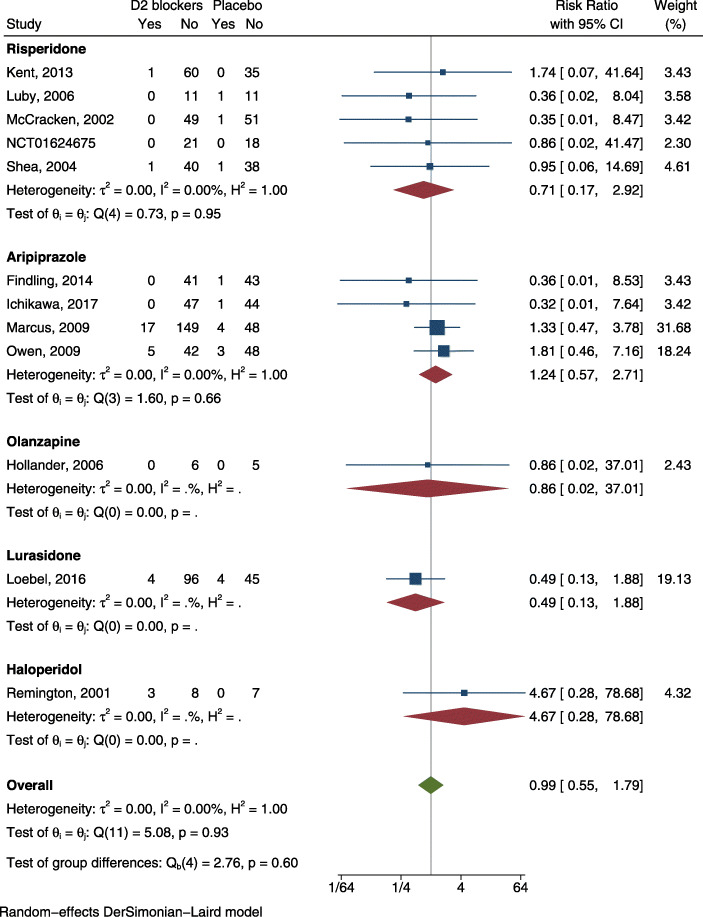
Forest plots for discontinuation due to adverse events

We found information on discontinuation due to any cause in 15 RCTs [33–47], and data on discontinuation due to adverse events in 12 RCTs [34–41, 44–47]. We evaluated the risk of bias for the included RCTs (Additional file 4). We show forest plots of selected outcomes in Figs. 2 and 3. Based on visual inspection of funnel plots, we considered that publication bias was not likely (Additional file 7). Results of meta-analyses and certainty of evidence in effect sizes are reported in Table 1.

**Table 1:** Summary of Findings for the comparison antipsychotics versus no antipsychotics: quantitative outcomes for acceptability

Antipsychotics versus no Antipsychotics for children and adolescents with ASD						
**Patient or Population**: children and adolescents with ASD **Setting**: Inpatients and Outpatients **Intervention**: Antipsychotics **Control**: no Antipsychotics						
Outcomes	**Anticipated absolute effects**^a^ (95% CI)		Relative effect (95% CI)	№ of participants (studies)	Certainty of the evidence (GRADE)	Comments
	**Risk with no Antipsychotics**	**Risk with Antipsychotics**				
Discontinuation due to any cause (Follow up: median 8 weeks)	244 per 1.000	**149 per 1.000** (117 to 190)	**RR 0.61** (0.48 to 0.78)	1124 (15 RCT) ^[33–47]^	⨁⨁⨁◯ MODERATE ^b^	Antipsychotics probably reduce the risk of dropout due to any cause
Discontinuation due to adverse events (Follow up: median 8 weeks)	39 per 1.000	**39 per 1.000** (22 to 70)	**RR 0.99** (0.55 to 1.79)	1010 (12 RCT) ^[34–47]^	⨁⨁◯◯ LOW ^a,b^	Antipsychotics may result in little to no difference in the risk of dropout due to adverse events

^a^The risk in the intervention group (and its 95% confidence interval) is based on the assumed risk in the comparison group and the relative effect of the intervention (and its 95% CI)

*CI* Confidence interval, *SMD* Standardised mean difference, *RR* Risk ratio

GRADE Working Group grades of evidence

High certainty: We are very confident that the true effect lies close to that of the estimate of the effect

Moderate certainty: We are moderately confident in the effect estimate: The true effect is likely to be close to the estimate of the effect, but there is a possibility that it is substantially different

Low certainty: Our confidence in the effect estimate is limited: The true effect may be substantially different from the estimate of the effect

Very low certainty: We have very little confidence in the effect estimate: The true effect is likely to be substantially different from the estimate of effect

Explanations

a. Downgraded by one level because the 95%CI for SMD goes from considerable beneficial effects to undesirable effects

b. Downgraded by one level because most studies showed an unclear risk for selection bias, four studies were at high risk for attrition bias, one study was at high risk for selection bias and one study was at high risk for reporting bias

References [33–47]

#### Actual use of antipsychotics in children and adolescents with ASD and predictive factors of their use

Jobski et al. (2017) [54] carried out a systematic review on the use of psychotropic drugs in individuals with ASD, identifying 47 studies that referred to a total of more than 300,000 individuals. In 15 of the 35 studies included in the review, antipsychotics were the most widely administered drugs. About 46% of the children considered across studies were taking psychotropic drugs, with a median prevalence of antipsychotic use of 17%.

About polypharmacological therapy, 22% of the children were taking several psychiatric drugs at the same time; The use of psychopharmacological products increased with age. The authors hypothesized some underlying causes for explaining this phenomenon: a) the development, during growth, of problem behaviors accompanied by an increase in physical strength; b) less reluctance to administer drugs by doctors and parents; (c) having already exhausted attempts at alternative treatments such as behavioral therapies. In addition, the authors noted a trend to switch from stimulants to antipsychotics and antidepressants as age increased, and this trend was attributed to the decrease in symptoms typical of comorbid Attention Deficit/Hyperactivity Disorder, alongside the onset of other problem behaviors such as anxiety, aggression, and depression. Another study [49], conducted in the UK and not included in Jobski et al. (2017) systematic review [54], enrolled a cohort of 3482 children and adolescents with ASD; according to the authors, about 10% of the included population was using antipsychotics, mainly risperidone (55%) and aripiprazole (32%), always associated with psychosocial therapy. The authors identified the following *socio-demographic predictors* of the use of antipsychotics: adolescent age (OR 1.11, 95% CI 1.05 to 1.16), low adaptive functioning inferred from the CGAS scale (OR 0.96, 95% CI 0.95 to 0.97), aggressive and self-harm behaviors (OR 1.85, 95% CI 1.30 to 2.63) and parental concern for symptoms (OR 2.02, 95% CI 1.27 to 3.22). *Clinical predictors* included hyperactivity (OR 1.44, 95% CI 1.01 to 2.06), depression (OR 2.36, 95% CI 1.37 to 4.09), obsessive-compulsive symptoms (OR 2).31, CI 95% from 1.16 to 4.61), tics (OR 2.76, CI 95% from 1.09 to 6.95), intellectual disability (OR 1.68, CI 95% from 1.11 to 2.53), and obviously psychosis (OR 5.71, CI 95% from 3.3 to 10.6).

### Feasibility

In this section, we summarized barriers and facilitators to the implementation and sustainability of antipsychotics administration using the findings from the three included studies [51–53]. We did not find studies that included children with ASD, but we found three studies considering children with intellectual disabilities and decided to use this evidence to inform this domain.

Among *facilitators*, the authors identified:

1. Nursing team. Nurses facilitate the monitoring of side effects and routine laboratory controls;
2. Electronic medical records. Electronic medical records are useful tools to assess treatment effects, monitor side effects, and facilitate communication between doctors;
3. Parental or caregiver support.

Among *barriers*, the authors identified:

1. Electronic medical records. Although perceived by some as facilitators, they are also considered a burden. There is no possibility to follow some parameters of treatment effects and side effects. When the system offers the possibility to monitor specific symptoms or effects of treatment, the possibilities are inflexible and time-consuming;
2. Workloads. The pressure and workloads experienced by health care professionals are barriers to the implementation of antipsychotics;
3. Cost barriers for the choice of drug (e.g. first-generation antipsychotics vs. second-generation antipsychotics);
4. Poor monitoring of metabolic adverse events of antipsychotics.

In Table 2, we summarized the results for all the considered EtD criteria.

**Table 2:** Summary of Findings for each Evidence to Decision (EtD) framework criterion

EtD Domain	Results
*Equity*	*No included studies*
*Acceptability*	*4 studies (2 cross-sectional studies* [48, 50]*, 1 cohort study* [49]*, 1 systematic review* [54]*) included in the qualitative synthesis, 3 of which specific to both our study population and intervention* [49, 50, 54]*, one only for the study population* [48]. *Antipsychotics were among the most prescribed drugs, with a median prevalence of use of 17%. A trend to switch from stimulants to anti-psychotics and anti-depressants as age increased was identified* [54]*.* *Socio-demographic predictors of the use of antipsychotics in our population: adolescent age, low adaptive functioning, aggressive and self-harm behaviors, and parental concern for symptoms. Clinical predictors of use: hyperactivity, depression, obsessive-compulsive symptoms, tics, intellectual disability, psychosis* [49]*.* *Drug therapy was the most frequently interrupted treatment (20%), mainly due to a lack of efficacy and AEs.* *Parents considered as crucial in choosing the treatment: opinions about the causes of the ASD, parental style, lifestyle, socio-economic status, ease of access to services and care, the impact of the media, and the testimonies of other families, but not scientific evidence* [48]*.* *Low income, child’s IQ ≥ 45, lower parents’ education correlated to poor satisfaction with the number of visits, learning tests, and behavioral assessment, respectively, in an RCT of risperidone* vs. *placebo* [50]*.* *Quantitative synthesis: antipsychotics in children and adolescents with ASD are acceptable (DO due to any cause: 15 RCTs* [33–47]*, RR 0.61, 95% CI 0.48–0.78, moderate certainty of evidence) and well tolerated (DO due to AEs: 12 RCTs* [34–41, 44–47]*, RR 0.99, 95% CI 0.55–1.79, low certainty of evidence).*
*Feasibility*	*3 cross-sectional studies* [51–53] *investigated the feasibility of administering antipsychotics to the general population (indirect evidence).* *Facilitators: Nursing team, Electronic medical records, Parental or caregiver support.* *Barriers: Electronic medical records, workloads, Cost barriers for the choice of drug, inadequate monitoring of metabolic AEs.*

*AE* Adverse events, *ASD* Autism Spectrum Disorder, *DO* Dropout, *EtD* Evidence to Decision, *RCT* Randomized controlled trial

## Discussion

Antipsychotics are among the most widely prescribed drugs in children and adolescents with ASD, with a median prevalence of use of 17% [54]. This is in line with both the prevalence of irritability in this population, for which risperidone and aripiprazole are effective and indicated [12, 55–58], and the frequent comorbidity with schizophrenia spectrum disorders symptoms [59].

On the basis of the available evidence, we found that antipsychotics were likely acceptable, and their implementation was potentially feasible. We did not find any information in the literature regarding the relation between antipsychotics administration and health equity. This could be because health disparities have not been observed, not expected, or not explored.

Parents reported the most frequent causes of drug discontinuation to be lack of efficacy and adverse events [48]. The higher rate of adverse events shown by recent meta-analyses [15, 55] did not impact on discontinuation due to adverse events in our study. Moreover, according to a recent meta-analysis [60], antipsychotics had a reduced discontinuation due to lack of efficacy when compared against placebo. The reduced discontinuation may partially explain also the observed strong protective effect of the drugs on dropouts due to any cause.

We did not find any study focusing specifically on the feasibility of antipsychotics administration in children and adolescents with ASD. However, while no specific barriers seemed to arise from the analysis of the acceptability, some concerns about the feasibility of proper monitoring of adverse events remained when analyzing indirect evidence [51–53, 61]. The implementation of facilitators could help provide better monitoring and solve drug-related problems [51, 53, 61, 62]. We found no evidence for the equity.

### Strengths and limitations

The conduct of evidence synthesis of contextual evidence that influences recommendations is an emerging field in systematic review research, and we believe that our attempt to provide such evidence is a strength. To inform each considered criterion of the EtD framework, we ran a comprehensive search strategy to retrieve both randomized and non-randomized evidence. We also performed a meta-analysis of randomized controlled trials to assess the acceptability and tolerability of antipsychotics for children and adolescents with ASD. In our opinion, the combination of quantitative and qualitative synthesis is an added study strength.

The use of the EtD framework in general, and the evaluation of its domains relating to equity, acceptability and feasibility, requires the panel to be familiar with the tool [63], and this is a potential limitation for the process of developing recommendations. To overcome this potential limitation, about 2 months before the presentation of the body of evidence on antipsychotics, an EtD framework on a pilot question was presented to the panel [64, 65]. In other experiences, panel members have reported that, when familiarity with the EtD framework is achieved, the tool helped them in structuring discussion, saving time, ensuring systematicity in the process of recommendation formulation [66].

Available evidence on acceptability, equity, and feasibility of antipsychotics in children and adolescents with ASD needs to be evaluated by the panel together with evidence on efficacy, safety (D’Alò GL, De Crescenzo F, Amato L, et al; ISACA guideline working group. Impact of antipsychotics in children and adolescents with autism spectrum disorder: a systematic review and meta-analysis, forthcoming), resources required and cost-effectiveness in formulating a recommendation.

We have not prospectively registered on PROSPERO or other databases the protocol for our systematic review, and this is a study limitation. However, the clinical question was formulated by a multidisciplinary panel of experts, and the methodology followed for the development of the systematic review was based on the manual developed and published by the ISS [18, 67].

The results of a previously ongoing trial (NCT00198107) [68] were published on clinicaltrial.gov after the recommendation formulated by the panel and based on the present systematic review was submitted to public consultation. However, data were consistent with the results of our study for both “Discontinuation due to any cause” (RR 0.51, CI 0.14 to 1.91), and “Discontinuation due to adverse events” (RR 1.02, CI 0.07 to 15.83).

## Conclusions

Antipsychotics in children and adolescents with ASD were likely acceptable and possibly feasible. We did not find evidence of concern for equity. Future clinical research needs to prioritize acceptable and feasible interventions that contribute to reducing health inequities [69].

Preferred reporting items for systematic reviews and meta-analyses (PRISMA) checklist is reported on Additional file 8.

## Supplementary Information

**Additional file 1.** Search strategy and results.

**Additional file 2.** Full methodology for data extraction.

**Additional file 3.** References for included and excluded studies, with reasons.

**Additional file 4.** Risk of bias summary.

**Additional file 5.** Newcastle Ottawa Scale (NOS).

**Additional file 6.** AMSTAR 2.

**Additional file 7.** Funnel plots.

**Additional file 8.** PRISMA checklist.

## Data Availability

All data supporting our findings is contained within the manuscript and the additional files. The authors and can be contacted at f.decrescenzo@deplazio.it. (FDC) for further clarification, if required.

## Abbreviations

ASD: Autism spectrum disorder
D2: Dopamine 2
EtD: Evidence to Decision
GRADE: grading of recommendations assessment, development and evaluation
ISS: Italian national institute of health
NOS: Newcastle Ottawa Scale
PRISMA: Preferred reporting items for systematic reviews and meta-analyses
