# Supplementary material for: Acceptability, equity, and feasibility of using antipsychotics in children and adolescents with autism spectrum disorder: a systematic review
Source: BMC Psychiatry. 2020 Nov 25;20:561. doi: 10.1186/s12888-020-02956-8 (PMC7687819; doi:10.1186/s12888-020-02956-8)
Supplement: Supplementary file 1 — Additional file 1. Search strategy and results. [file 12888_2020_2956_MOESM1_ESM.docx]

Additional file 1.

# Search Strategy for observational studies on Equity, Acceptability, Feasibility.

## 1. MEDLINE search strategy

**MEDLINE (Ovid MEDLINE® Epub Ahead of Print, In-Process & Other Non-Indexed Citations, Ovid MEDLINE® Daily and Ovid MEDLINE®) 1946 to January 22, 2019**

1. exp Child Development Disorders, Pervasive/
2. (autis* or ASD or ASDs or PDD or PDDs).tw.
3. pervasive developmental disorder$.tw.
4. asperg$.tw.
5. kanner$.tw.
6. 1 or 2 or 3 or 4 or 5
7. exp child/ or adolescent/ or pediatrics/
8. (child* or schoolchild* or kid or kids or toddler* or adoles* or teen*or boy* or girl* or minors* or underag* or under age or juvenil* or youth* or kindergar* or puberty or pubescen* or prepubescen* or prepuberty* or pediatric* or paediatric* or peadiatric* or preschool* or schoolage).tw.
9. (school adj2 age*).ti,ab.
10. 7 or 8 or 9
11. 6 and 10
12. exp Antipsychotic Agents/
13. exp BUTYROPHENONES/
14. exp PHENOTHIAZINES/
15. exp THIOXANTHENES/
16. (anti psychotic* or antipsychotic*).mp.
17. amisulprid$.mp.
18. aripiprazol*.mp.
19. asenapin*.mp.
20. Blonanserin.mp.
21. Chlorpromazin*.mp.
22. Chlorprothixen*.mp.
23. Clotiapin*.mp.
24. Clozapin*.mp.
25. Droperidol.mp.
26. Flupentixol.mp.
27. Fluphenazin*.mp.
28. Haloperidol.mp.
29. Iloperidon*.mp.
30. Levomepromazin*.mp.
31. Loxapin*.mp.
32. Lurasidon*.mp.
33. Melperon*.mp.
34. Mesoridazin*.mp.
35. Molindon*.mp.
36. Mosapramin*.mp.
37. Olanzapin*.mp.
38. Paliperidon*.mp.
39. Periciazin*.mp.
40. Perospiron*.mp.
41. Pimozid*.mp.
42. Prochlorperazin*.mp.
43. Promazin*.mp.
44. Quetiapin*.mp.
45. Remoxiprid*.mp.
46. Risperidon*.mp.
47. Sertindol*.mp.
48. Sulpirid*.mp.
49. Thioproperazin*.mp.
50. Thioridazin*.mp.
51. Thiothixen*.mp.
52. Tiaprid*.mp.
53. Trifluoperazin*.mp.
54. Ziprasidon*.mp.
55. Zotepin*.mp.
56. Zuclopenthixol.mp.
57. Mesoridazin*.mp.
58. 12 or 13 or 14 or 15 or 16 or 17 or 18 or 19 or 20 or 21 or 22 or 23 or 24 or 25 or 26 or 27 or 28 or 29 or 30 or 31 or 32 or 33 or 34 or 35 or 36 or 37 or 38 or 39 or 40 or 41 or 42 or 43 or 44 or 45 or 46 or 47 or 48 or 49 or 50 or 51 or 52 or 53 or 54 or 55 or 56 or 57
59. 11 and 58
60. *Attitude to Health/
61. *Patient Participation/
62. *Patient Preference/
63. (choice or choices).ti.
64. value*.ti.
65. health state values.ti,ab.
66. valuation*.ti.
67. expectation*.ti,ab.
68. attitude*.ti,ab.
69. acceptab*.ti,ab.
70. point of view.ti,ab.
71. patient* participation.ti,ab.
72. user* participation.ti,ab.
73. user* perspective*.ti,ab.
74. patient* perce*.ti,ab.
75. user* perce*.ti,ab.
76. user view*.ti,ab.
77. patient* view*.ti,ab.
78. 60 or 61 or 62 or 63 or 64 or 65 or 66 or 67 or 68 or 69 or 70 or 71 or 72 or 73 or 74 or 75 or 76 or 87
79. 59 and 78

## 2. EMBASE search strategy

**EMBASE (via embase.com)**

**Database: Embase Classic + Embase, 1947 to 2018 October 26.**

1. (autis* or ASD or ASDs).ti,ab.
2. PDD.ti,ab.
3. pervasive developmental disorder$.tw.
4. kanner$.tw.
5. (asperg$ not aspergill$).tw.
6. 1 or 2 or 3 or 4 or 5
7. exp child/ or adolescent/ or pediatrics/
8. (child* or schoolchild* or kid or kids or toddler* or adoles* or teen*or boy* or girl* or minors* or underag* or under age or juvenil* or youth* or kindergar* or puberty or pubescen* or prepubescen* or prepuberty* or pediatric* or paediatric* or peadiatric* or preschool* or schoolage).tw.
9. (school adj2 age*).ti,ab.
10. 7 or 8 or 9
11. 6 and 10
12. exp clinical trial/
13. exp crossover procedure/
14. exp double blind procedure/
15. exp controlled clinical trial/
16. (placebo or assign* or allocat* or volunteer* or random* or factorial* or crossover).ti,ab.
17. ((singl$ or doubl$ or trebl$ or tripl$) adj3 (blind$ or mask$)).tw.
18. 12 or 13 or 14 or 15 or 16 or 17
19. exp neuroleptic agent/
20. (anti psychotic* or antipsychotic*).mp.
21. amisulprid$.mp.
22. aripiprazol*.mp.
23. asenapin*.mp.
24. Blonanserin.mp.
25. Chlorpromazin*.mp.
26. Chlorprothixen*.mp.
27. Clotiapin*.mp.
28. Clozapin*.mp.
29. Droperidol.mp.
30. Flupentixol.mp.
31. Fluphenazin*.mp.
32. Haloperidol.mp.
33. Iloperidon*.mp.
34. Levomepromazin*.mp.
35. Loxapin*.mp.
36. Lurasidon*.mp.
37. Melperon*.mp.
38. Mesoridazin*.mp.
39. Molindon*.mp.
40. Mosapramin*.mp.
41. Olanzapin*.mp.
42. Paliperidon*.mp.
43. Periciazin*.mp.
44. Perospiron*.mp.
45. Pimozid*.mp.
46. Prochlorperazin*.mp.
47. Promazin*.mp.
48. Quetiapin*.mp.
49. Remoxiprid*.mp.
50. Risperidon*.mp.
51. Sertindol*.mp.
52. Sulpirid*.mp.
53. Thioproperazin*.mp.
54. Thioridazin*.mp.
55. Thiothixen*.mp.
56. Tiaprid*.mp.
57. Trifluoperazin*.mp.
58. Ziprasidon*.mp.
59. Zotepin*.mp.
60. Zuclopenthixol.mp.
61. Mesoridazin*.mp.
62. 19 or 20 or 21 or 22 or 23 or 24 or 25 or 26 or 27 or 28 or 29 or 30 or 31 or 32 or 33 or 34 or 35 or 36 or 37 or 38 or 39 or 40 or 41 or 42 or 43 or 44 or 45 or 46 or 47 or 48 or 49 or 50 or 51 or 52 or 53 or 54 or 55 or 56 or 57 or 58 or 59 or 60 or 61
63. 11 and 18 and 62
64. *attitude to health/
65. *patient participation/
66. *patient preference/
67. (choice or choices).ti.
68. value*.ti.
69. health state values.ti,ab.
70. valuation*.ti.
71. expectation*.ti,ab.
72. attitude*.ti,ab.
73. acceptab*.ti,ab.
74. point of view.ti,ab.
75. patient* participation.ti,ab.
76. user* participation.ti,ab.
77. user* perspective*.ti,ab.
78. patient* perce*.ti,ab.
79. user* perce*.ti,ab.
80. user view*.ti,ab.
81. patient* view*.ti,ab.
82. 64 or 65 or 66 or 67 or 68 or 69 or 70 or 71 or 72 or 73 or 74 or 75 or 76 or 77 or 78 or 79 or 80 or 81
83. 11 and 62 and 82

## 3. PsycINFO search strategy

**PsycINFO (via EBSCO HOST) 1806 to present**

1. (autis* or ASD or ASDs).ti,ab.
2. PDD.ti,ab.
3. pervasive developmental disorder$.tw.
4. kanner$.tw.
5. (asperg$ not aspergill$).tw.
6. 1 or 2 or 3 or 4 or 5
7. exp child/ or adolescent/ or pediatrics/
8. (child* or schoolchild* or kid or kids or toddler* or adoles* or teen*or boy* or girl* or minors* or underag* or under age or juvenil* or youth* or kindergar* or puberty or pubescen* or prepubescen* or prepuberty* or pediatric* or paediatric* or peadiatric* or preschool* or schoolage).tw. (894990)
9. (school adj2 age*).ti,ab.
10. 7 or 8 or 9
11. 6 and 10
12. exp clinical trial/
13. (placebo or assign* or allocat* or volunteer* or random* or factorial* or crossover).ti,ab.
14. ((singl$ or doubl$ or trebl$ or tripl$) adj3 (blind$ or mask$)).tw.
15. (anti psychotic* or antipsychotic*).mp.
16. amisulprid$.mp.
17. aripiprazol*.mp.
18. asenapin*.mp.
19. Blonanserin.mp.
20. Chlorpromazin*.mp.
21. Chlorprothixen*.mp.
22. Clotiapin*.mp.
23. Clozapin*.mp.
24. Droperidol.mp.
25. Flupentixol.mp.
26. Fluphenazin*.mp.
27. Haloperidol.mp.
28. Iloperidon*.mp.
29. Levomepromazin*.mp.
30. Loxapin*.mp.
31. Lurasidon*.mp.
32. Melperon*.mp.
33. Mesoridazin*.mp.
34. Molindon*.mp.
35. Mosapramin*.mp.
36. Olanzapin*.mp.
37. Paliperidon*.mp.
38. Periciazin*.mp.
39. Perospiron*.mp.
40. Pimozid*.mp.
41. Prochlorperazin*.mp.
42. Promazin*.mp.
43. Quetiapin*.mp.
44. Remoxiprid*.mp.
45. Risperidon*.mp.
46. Sertindol*.mp.
47. Sulpirid*.mp.
48. Thioproperazin*.mp.
49. Thioridazin*.mp.
50. Thiothixen*.mp.
51. Tiaprid*.mp.
52. Trifluoperazin*.mp.
53. Ziprasidon*.mp.
54. Zotepin*.mp.
55. Zuclopenthixol.mp.
56. Mesoridazin*.mp.
57. *patient participation/
58. (choice or choices).ti.
59. value*.ti.
60. health state values.ti,ab.
61. valuation*.ti.
62. expectation*.ti,ab.
63. attitude*.ti,ab.
64. acceptab*.ti,ab.
65. point of view.ti,ab.
66. patient* participation.ti,ab.
67. user* participation.ti,ab.
68. user* perspective*.ti,ab.
69. patient* perce*.ti,ab.
70. user* perce*.ti,ab.
71. user view*.ti,ab.
72. patient* view*.ti,ab.
73. patient preference.mp.
74. 12 or 13 or 14
75. 15 or 16 or 17 or 18 or 19 or 20 or 21 or 22 or 23 or 24 or 25 or 26 or 27 or 28 or 29 or 30 or 31 or 32 or 33 or 34 or 35 or 36 or 37 or 38 or 39 or 40 or 41 or 42 or 43 or 44 or 45 or 46 or 47 or 48 or 49 or 50 or 51 or 52 or 53 or 54 or 55 or 56
76. 57 or 58 or 59 or 60 or 61 or 62 or 63 or 64 or 65 or 66 or 67 or 68 or 69 or 70 or 71 or 72 or 73
77. 11 and 74 and 75
78. 11 and 74 and 76

We searched for ongoing clinical trials and unpublished trials via Internet searches on the following web-sites:

- ClinicalTrials.gov ([www.clinicaltrials.gov](http://www.clinicaltrials.gov/));
- World Health Organization (WHO) International Clinical Trials Registry Platform (ICTRP) ([apps.who.int/trialsearch/](http://apps.who.int/trialsearch/)).
- Australian New Zealand Clinical Trial Registry (ANZCTR) (anzctr.org.au/BasicSearch.aspx)

## Numbers of citations by each database

| **Databases, trial registers and other sources** | **Citations** |
| --- | --- |
| **Databases:** |  |
| PubMed | 10 |
| Embase | 38 |
| PsycINFO | 109 |
| **Total (databases)** | **157** |
| Duplicate records removed | 30 |
| **Total (databases) after duplicates removal** | **127** |
|  |  |
| **Trial registers:** |  |
| ClinicalTrials.gov | 0 |
| World Health Organization (WHO) International Clinical Trials Registry Platform (ICTRP) ([apps.who.int/trialsearch/](http://apps.who.int/trialsearch/)). | 0 |
| Australian New Zealand Clinical Trial Registry (ANZCTR) (anzctr.org.au/BasicSearch.aspx) | 0 |
| **Total (trial registers)** | **0** |
|  |  |
| **Other sources:** | **1** |
|  |  |
| **Total citations:** | **128** |

# Search Strategy and Results for Randomized Controlled Trials.

## 1. CENTRAL search strategy

**CENTRAL (via onlinelibrary.wiley.com)**

1. MeSH descriptor: [Child Development Disorders, Pervasive] explode all trees
2. asperger*
3. (autis* or ASD or ASDs):ti,ab,kw
4. kanner*
5. ((pervasiv* NEXT development* NEXT disorder*) OR PDD or PDDs):ti,ab,kw
6. #1 or #2 or #3 or #4 # or #5
7. MeSH descriptor: [Antipsychotic Agents] explode all trees
8. MeSH descriptor: [Butyrophenones] explode all trees
9. MeSH descriptor: [Phenothiazines] explode all trees
10. MeSH descriptor: [Thioxanthenes] explode all trees
11. (anti next psychotic*) or antipsychotic*:ti,ab,kw
12. Amisulprid*
13. Aripiprazol*
14. Asenapin*
15. Blonanserin
16. Chlorpromazin*
17. Chlorprothixen*
18. Clotiapin*
19. Clozapin*
20. Droperidol
21. Flupentixol
22. Fluphenazin*
23. Haloperidol
24. Iloperidon*
25. Levomepromazin*
26. Loxapin*
27. Lurasidon*
28. Melperon*
29. Mesoridazin*
30. Molindon*
31. Mosapramin*
32. Olanzapin*
33. Paliperidon*
34. Periciazin*
35. Perospiron*
36. Pimozid*
37. Prochlorperazin*
38. Promazin*
39. Quetiapin*
40. Remoxiprid*
41. Risperidon*
42. Sertindol*
43. Sulpirid*
44. Thioproperazin*
45. Thioridazin*
46. Thiothixen*
47. Tiaprid*
48. Trifluoperazin*
49. Ziprasidon*
50. Zotepin*
51. Zuclopenthixol
52. {or #7-#51}
53. #6 and #52
54. MeSH descriptor: [Adolescent] explode all trees
55. MeSH descriptor: [Adolescent Medicine] explode all trees
56. MeSH descriptor: [Child] explode all trees
57. MeSH descriptor: [Minors] explode all trees
58. MeSH descriptor: [Pediatrics] explode all trees
59. MeSH descriptor: [Young Adult] explode all trees
60. (child* or schoolchild* or kid or kids or toddler* or adoles* or teen*or boy* or girl* or minors* or underag* or under age or juvenil* or youth* or kindergar* or puberty or pubescen* or prepubescen* or prepuberty* or pediatric* or paediatric* or peadiatric* or preschool* or schoolage):ti,ab,kw
61. ((grade next school*) or (pre next school*) or (school next age*) or schoolchild*):ti,ab,kw
62. ((colleg* or highschool* or school* or universit*) near/2 (age* or student*)):ti,ab,kw
63. (young* next (adult* or men or mens or people* or person* or women*))
64. #54 or #55 or #56 or #57 or #58 or #59 or #60 or #61 or #62 or #63
65. #53 AND #64

## 2. MEDLINE search strategy

**MEDLINE (Ovid MEDLINE® Epub Ahead of Print, In-Process & Other Non-Indexed Citations, Ovid MEDLINE® Daily and Ovid MEDLINE®) 1946 to January 22, 2019**

1. exp Child Development Disorders, Pervasive/
2. (autis* or ASD or ASDs or PDD or PDDs).tw.
3. pervasive developmental disorder$.tw.
4. asperg$.tw.
5. kanner$.tw.
6. 1 or 2 or 3 or 4 or 5
7. exp child/ or adolescent/ or pediatrics/
8. (child* or schoolchild* or kid or kids or toddler* or adoles* or teen*or boy* or girl* or minors* or underag* or under age or juvenil* or youth* or kindergar* or puberty or pubescen* or prepubescen* or prepuberty* or pediatric* or paediatric* or peadiatric* or preschool* or schoolage).tw.
9. (school adj2 age*).ti,ab.
10. 7 or 8 or 9
11. 6 and 10
12. exp Antipsychotic Agents/
13. exp BUTYROPHENONES/
14. exp PHENOTHIAZINES/
15. exp THIOXANTHENES/
16. (anti psychotic* or antipsychotic*).mp.
17. amisulprid$.mp.
18. aripiprazol*.mp.
19. asenapin*.mp.
20. Blonanserin.mp.
21. Chlorpromazin*.mp.
22. Chlorprothixen*.mp.
23. Clotiapin*.mp.
24. Clozapin*.mp.
25. Droperidol.mp.
26. Flupentixol.mp.
27. Fluphenazin*.mp.
28. Haloperidol.mp.
29. Iloperidon*.mp.
30. Levomepromazin*.mp.
31. Loxapin*.mp.
32. Lurasidon*.mp.
33. Melperon*.mp.
34. Mesoridazin*.mp.
35. Molindon*.mp.
36. Mosapramin*.mp.
37. Olanzapin*.mp.
38. Paliperidon*.mp.
39. Periciazin*.mp.
40. Perospiron*.mp.
41. Pimozid*.mp.
42. Prochlorperazin*.mp.
43. Promazin*.mp.
44. Quetiapin*.mp.
45. Remoxiprid*.mp.
46. Risperidon*.mp.
47. Sertindol*.mp.
48. Sulpirid*.mp.
49. Thioproperazin*.mp.
50. Thioridazin*.mp.
51. Thiothixen*.mp.
52. Tiaprid*.mp.
53. Trifluoperazin*.mp.
54. Ziprasidon*.mp.
55. Zotepin*.mp.
56. Zuclopenthixol.mp.
57. Mesoridazin*.mp.
58. 12 or 13 or 14 or 15 or 16 or 17 or 18 or 19 or 20 or 21 or 22 or 23 or 24 or 25 or 26 or 27 or 28 or 29 or 30 or 31 or 32 or 33 or 34 or 35 or 36 or 37 or 38 or 39 or 40 or 41 or 42 or 43 or 44 or 45 or 46 or 47 or 48 or 49 or 50 or 51 or 52 or 53 or 54 or 55 or 56 or 57
59. 11 and 58
60. randomized controlled trial.pt.
61. controlled clinical trial.pt.
62. randomi#ed.ab.
63. placebo$.ab.
64. drug therapy.fs.
65. randomly.ab.
66. trial.ab.
67. groups.ab.
68. 60 or 61 or 62 or 63 or 64 or 65 or 66 or 67
69. 59 and 68

## 3. EMBASE search strategy

**EMBASE (via embase.com)**

**Database: Embase Classic + Embase, 1947 to 2018 October 26.**

1. (autis* or ASD or ASDs).ti,ab.
2. PDD.ti,ab.
3. pervasive developmental disorder$.tw.
4. kanner$.tw.
5. (asperg$ not aspergill$).tw.
6. 1 or 2 or 3 or 4 or 5
7. exp child/ or adolescent/ or pediatrics/
8. (child* or schoolchild* or kid or kids or toddler* or adoles* or teen*or boy* or girl* or minors* or underag* or under age or juvenil* or youth* or kindergar* or puberty or pubescen* or prepubescen* or prepuberty* or pediatric* or paediatric* or peadiatric* or preschool* or schoolage).tw.
9. (school adj2 age*).ti,ab.
10. 7 or 8 or 9
11. 6 and 10
12. exp clinical trial/
13. exp crossover procedure/
14. exp double blind procedure/
15. exp controlled clinical trial/
16. (placebo or assign* or allocat* or volunteer* or random* or factorial* or crossover).ti,ab.
17. ((singl$ or doubl$ or trebl$ or tripl$) adj3 (blind$ or mask$)).tw.
18. 12 or 13 or 14 or 15 or 16 or 17
19. exp neuroleptic agent/
20. (anti psychotic* or antipsychotic*).mp.
21. amisulprid$.mp.
22. aripiprazol*.mp.
23. asenapin*.mp.
24. Blonanserin.mp.
25. Chlorpromazin*.mp.
26. Chlorprothixen*.mp.
27. Clotiapin*.mp.
28. Clozapin*.mp.
29. Droperidol.mp.
30. Flupentixol.mp.
31. Fluphenazin*.mp.
32. Haloperidol.mp.
33. Iloperidon*.mp.
34. Levomepromazin*.mp.
35. Loxapin*.mp.
36. Lurasidon*.mp.
37. Melperon*.mp.
38. Mesoridazin*.mp.
39. Molindon*.mp.
40. Mosapramin*.mp.
41. Olanzapin*.mp.
42. Paliperidon*.mp.
43. Periciazin*.mp.
44. Perospiron*.mp.
45. Pimozid*.mp.
46. Prochlorperazin*.mp.
47. Promazin*.mp.
48. Quetiapin*.mp.
49. Remoxiprid*.mp.
50. Risperidon*.mp.
51. Sertindol*.mp.
52. Sulpirid*.mp.
53. Thioproperazin*.mp.
54. Thioridazin*.mp.
55. Thiothixen*.mp.
56. Tiaprid*.mp.
57. Trifluoperazin*.mp.
58. Ziprasidon*.mp.
59. Zotepin*.mp.
60. Zuclopenthixol.mp.
61. Mesoridazin*.mp.
62. 19 or 20 or 21 or 22 or 23 or 24 or 25 or 26 or 27 or 28 or 29 or 30 or 31 or 32 or 33 or 34 or 35 or 36 or 37 or 38 or 39 or 40 or 41 or 42 or 43 or 44 or 45 or 46 or 47 or 48 or 49 or 50 or 51 or 52 or 53 or 54 or 55 or 56 or 57 or 58 or 59 or 60 or 61
63. 11 and 18 and 62

## 4. Web of Science search strategy

**WOS (via THOMSON REUTERS)**

Indexes=SCI-EXPANDED, SSCI, A&HCI, CPCI-S, CPCI-SSH, ESCI Timespan=All years

1. TI=(autis* or asperger* or "pervasive developmental " or (pervasive NEAR/3 child))TS=(omega 3 or omega 6)
2. TS=(anti psychotic* OR antipsychotic* OR amisulprid* OR aripiprazol* OR asenapin* OR blonanserin OR chlorpromazin* OR chlorprothixen* OR clotiapin* OR clozapin* OR droperidol OR flupentixol OR fluphenazin* OR haloperidol OR iloperidon* OR levomepromazin* OR loxapin* OR lurasidon* OR melperon* OR mesoridazin* OR molindon* OR mosapramin* OR olanzapin* OR paliperidon* OR periciazin* OR perospiron* OR pimozid* OR prochlorperazin* OR promazin* OR quetiapin* OR remoxiprid* OR risperidon* OR sertindol* OR sulpirid* OR thioproperazin* OR thioridazin* OR thiothixen* OR tiaprid* OR trifluoperazin* OR ziprasidon* OR zotepin* OR zuclopenthixol)
3. TS=(child* or schoolchild* or kid or kids or toddler* or adoles* or teen*or boy* or girl* or minors* or underag* or under age or juvenil* or youth* or kindergar* or puberty or pubescen* or prepubescen* or prepuberty* or pediatric* or paediatric* or peadiatric* or preschool* or schoolage)
4. #3 AND #2 AND #1
5. TS=(random* or control* or trial* or groups* or effectiveness or evaluation or placebo*)
6. #5 AND #4

## 5. PsycINFO search strategy

**PsycINFO (via EBSCO HOST) 1806 to present**

1. (autis* or ASD or ASDs).ti,ab.
2. PDD.ti,ab.
3. pervasive developmental disorder$.tw.
4. kanner$.tw.
5. (asperg$ not aspergill$).tw.
6. 1 or 2 or 3 or 4 or 5
7. exp child/ or adolescent/ or pediatrics/
8. (child* or schoolchild* or kid or kids or toddler* or adoles* or teen*or boy* or girl* or minors* or underag* or under age or juvenil* or youth* or kindergar* or puberty or pubescen* or prepubescen* or prepuberty* or pediatric* or paediatric* or peadiatric* or preschool* or schoolage).tw.
9. (school adj2 age*).ti,ab.
10. 7 or 8 or 9
11. 6 and 10
12. exp clinical trial/
13. (placebo or assign* or allocat* or volunteer* or random* or factorial* or crossover).ti,ab.
14. ((singl$ or doubl$ or trebl$ or tripl$) adj3 (blind$ or mask$)).tw.
15. (anti psychotic* or antipsychotic*).mp.
16. amisulprid$.mp.
17. aripiprazol*.mp.
18. asenapin*.mp.
19. Blonanserin.mp.
20. Chlorpromazin*.mp.
21. Chlorprothixen*.mp.
22. Clotiapin*.mp.
23. Clozapin*.mp.
24. Droperidol.mp.
25. Flupentixol.mp.
26. Fluphenazin*.mp.
27. Haloperidol.mp.
28. Iloperidon*.mp.
29. Levomepromazin*.mp.
30. Loxapin*.mp.
31. Lurasidon*.mp.
32. Melperon*.mp.
33. Mesoridazin*.mp.
34. Molindon*.mp.
35. Mosapramin*.mp.
36. Olanzapin*.mp.
37. Paliperidon*.mp.
38. Periciazin*.mp.
39. Perospiron*.mp.
40. Pimozid*.mp.
41. Prochlorperazin*.mp.
42. Promazin*.mp.
43. Quetiapin*.mp.
44. Remoxiprid*.mp.
45. Risperidon*.mp.
46. Sertindol*.mp.
47. Sulpirid*.mp.
48. Thioproperazin*.mp.
49. Thioridazin*.mp.
50. Thiothixen*.mp.
51. Tiaprid*.mp.
52. Trifluoperazin*.mp.
53. Ziprasidon*.mp.
54. Zotepin*.mp.
55. Zuclopenthixol.mp.
56. Mesoridazin*.mp.
57. 12 or 13 or 14
58. 15 or 16 or 17 or 18 or 19 or 20 or 21 or 22 or 23 or 24 or 25 or 26 or 27 or 28 or 29 or 30 or 31 or 32 or 33 or 34 or 35 or 36 or 37 or 38 or 39 or 40 or 41 or 42 or 43 or 44 or 45 or 46 or 47 or 48 or 49 or 50 or 51 or 52 or 53 or 54 or 55 or 56
59. 11 and 57 and 58

We searched for ongoing clinical trials and unpublished trials via Internet searches on the following web-sites:

- ClinicalTrials.gov ([www.clinicaltrials.gov](http://www.clinicaltrials.gov/));
- World Health Organization (WHO) International Clinical Trials Registry Platform (ICTRP) ([apps.who.int/trialsearch/](http://apps.who.int/trialsearch/)).

## Numbers of citations by each database

| **Databases, trial registers and other sources** | **Citations** |
| --- | --- |
| **Databases:** |  |
| PubMed | 696 |
| Central | 215 |
| Web of Science | 418 |
| Embase | 492 |
| PsycINFO | 166 |
| **Total (databases)** | **1987** |
| Duplicate records removed | 779 |
| **Total (databases) after duplicates removed** | **1208** |
|  |  |
| **Trial registers:** |  |
| ClinicalTrials.gov | 52 |
| World Health Organization (WHO) International Clinical Trials Registry Platform (ICTRP) ([apps.who.int/trialsearch/](http://apps.who.int/trialsearch/)). | 0 |
| **Total (trial registers)** | **52** |
|  |  |
| **Other sources:** | **0** |
|  |  |
| **Total citations:** | **1260** |
