# Supplementary material for: Acceptability, equity, and feasibility of using antipsychotics in children and adolescents with autism spectrum disorder: a systematic review
Source: BMC Psychiatry. 2020 Nov 25;20:561. doi: 10.1186/s12888-020-02956-8 (PMC7687819; doi:10.1186/s12888-020-02956-8)
Supplement: Supplementary file 2 — Additional file 2. Full methodology for data extraction. [file 12888_2020_2956_MOESM2_ESM.docx]

Additional file 2.

**Full methodology for data extraction.**

**1) Equity:**

We extracted data from systematic reviews, randomized and non-randomized studies about the influence of socio-economic and cultural factors on the absolute effectiveness of D2 receptor blockers or the importance of the problem (2,3). Factors taken into account when assessing subgroup effects included the following: age, disability, sexual orientation, time-dependent situations, relationships, place of residence, race/ethnicity/culture, language, occupation, gender/sex, religion, education, socioeconomic status, or social capital) (1,3). We extracted both quantitative and qualitative data.

**2) Acceptability:**

We extracted narrative data from systematic reviews and primary studies (both randomized and non-randomised) to assess acceptability from any stakeholder (2), while we used discontinuation due to any cause and due to adverse events from RCTs only to evaluate the acceptability by children and adolescents with ASD.

**3) Feasibility:**

We inspected the background and discussion sections of the retrieved systematic reviews, non-randomized studies and RCTs to look for data and consideration on barriers and facilitators to the implementation of therapy and sustainability of the intervention (2).

**References:**

1. O’Neill J, Tabish H, Welch V, Petticrew M, Pottie K, Clarke M, et al. Applying an equity lens to interventions: using PROGRESS ensures consideration of socially stratifying factors to illuminate inequities in health. J Clin Epidemiol 2014;67:56e64.
2. Schünemann H, Brożek J, Guyatt G, Oxman A, editors. GRADE handbook for grading quality of evidence and strength of recommendations. Updated October 2013. The GRADE Working Group, 2013. Available fromguidelinedevelopment.org/handbook.
3. Welch VA, Akl EA, Guyatt G, Pottie K, Eslava-Schmalbach J, Ansari MT, et al. GRADE equity guidelines 1: considering health equity in GRADE guideline development: introduction and rationale. J Clin Epidemiol. 2017A;90:59-67
