# Supplementary material for: Acceptability, equity, and feasibility of using antipsychotics in children and adolescents with autism spectrum disorder: a systematic review
Source: BMC Psychiatry. 2020 Nov 25;20:561. doi: 10.1186/s12888-020-02956-8 (PMC7687819; doi:10.1186/s12888-020-02956-8)
Supplement: Supplementary file 3 — Additional file 3. References for included and excluded studies, with reasons. [file 12888_2020_2956_MOESM3_ESM.docx]

**Additional file 3**

# Full References for included and excluded studies from the search strategy for randomized controlled trials.

## Full References for included Randomized Controlled trials

1. Campbell 1978
   1. Campbell M, Anderson LT, Meier M, Cohen IL, Small AM, Samit C, Sachar EJ. A comparison of haloperidol and behavior therapy and their interaction in autistic children. J Am Acad Child Psychiatry. 1978 Autumn;17(4):640-55. PubMed PMID: 370186.
2. Findling 2014
   1. Findling RL, Mankoski R, Timko K, Lears K, McCartney T, McQuade RD, Eudicone JM, Amatniek J, Marcus RN, Sheehan JJ. A randomized controlled trial investigating the safety and efficacy of aripiprazole in the long-term maintenance treatment of pediatric patients with irritability associated with autistic disorder. J Clin Psychiatry. 2014 Jan;75(1):22-30. doi:10.4088/JCP.13m08500. PubMed PMID: 24502859.
   2. NCT01227668. Phase IV Long-term Maintenance Study of Aripiprazole in the Treatment of Irritability Associated With Autistic Disorder. First Posted: 25th Oct 2010. Accessed: 24th Jan 2019.
3. Hollander 2006
   1. Hollander E, Wasserman S, Swanson EN, et al. A double-blind placebo-controlled pilot study of olanzapine in childhood/adolescent pervasive developmental disorder. J Child Adolesc Psychopharmacol. 2006;16(5):541-8. doi:10.1089/cap.2006.16.541. PMID: 17069543.
4. Ichikawa 2017
   1. Ichikawa H, Mikami K, Okada T, Yamashita Y, Ishizaki Y, Tomoda A, Ono H, Usuki C, Tadori Y. Aripiprazole in the Treatment of Irritability in Children and Adolescents with Autism Spectrum Disorder in Japan: A Randomized, Double-blind, Placebo-controlled Study. Child Psychiatry Hum Dev. 2017 Oct;48(5):796-806. doi: 10.1007/s10578-016-0704-x. PubMed PMID: 28004215; PubMed Central PMCID: PMC5617873.
   2. Ichikawa H, Hiratani M, Yasuhara A, Tsujii N, Oshimo T, Ono H, Tadori Y. An open-label extension long-term study of the safety and efficacy of aripiprazole for irritability in children and adolescents with autistic disorder in Japan. Psychiatry Clin Neurosci. 2018 Feb;72(2):84-94. doi: 10.1111/pcn.12607. Epub 2017 Nov 8. PubMed PMID: 28941259.
   3. NCT01617460. A Long-term, Extended Treatment Study of Aripiprazole in Pediatric Patients With Autistic Disorder. First Posted: 12^th^ June 2012. Accessed: 23^rd^ Jan 2019.
5. Kent 2013
   1. Kent JM, Kushner S, Ning X, et al. Risperidone dosing in children and adolescents with autistic disorder: a double-blind, placebo-controlled study. J Autism Dev 197 Disord. 2013 Aug;43(8):1773-83. doi: 10.1007/s10803-012-1723-5. PMID: 23212807.
   2. NCT00576732. A Study of the Effectiveness and Safety of Two Doses of Risperidone in the Treatment of Children and Adolescents With Autistic Disorder. First Posted: 19^th^ December 2007. Accessed: 23^rd^ Jan 2019.
6. Loebel 2016
   1. Loebel A, Brams M, Goldman RS, et al. Lurasidone for the treatment of irritability with autistic disorder. J Autism Dev Disord. 2016;46:1153-63.
   2. NCT01911442. Lurasidone Pediatric Autism Study. First Posted: 30^th^ Jul 2013. Accessed: 3^rd^ Feb 2019.
7. Luby 2006
   1. Luby J, Mrakotsky C, Stalets MM, et al. Risperidone in preschool children with autistic spectrum disorders: an investigation of safety and efficacy. J Child Adoles Psychopharmacol. 2006;16(5):575-87. doi:10.1089/cap.2006.16.575. PMID: 17069546.
8. Marcus 2009
   1. Marcus RN, Owen R, Kamen L, Manos G, McQuade RD, Carson WH, Aman MG. A placebo-controlled, fixed-dose study of aripiprazole in children and adolescents with irritability associated with autistic disorder. J Am Acad Child Adolesc Psychiatry. 2009 Nov;48(11):1110-9. doi: 10.1097/CHI.0b013e3181b76658. PubMed PMID: 19797985.
   2. Marcus RN, Owen R, Manos G, Mankoski R, Kamen L, McQuade RD, Carson WH, Findling RL. Safety and tolerability of aripiprazole for irritability in pediatric patients with autistic disorder: a 52-week, open-label, multicenter study. J Clin Psychiatry. 2011 Sep;72(9):1270-6. doi: 10.4088/JCP.09m05933. Epub 2011 Jul 26. PubMed PMID: 21813076.
   3. Marcus RN, Owen R, Manos G, Mankoski R, Kamen L, McQuade RD, Carson WH, Corey-Lisle PK, Aman MG. Aripiprazole in the treatment of irritability in pediatric patients (aged 6-17 years) with autistic disorder: results from a 52-week, open-label study. J Child Adolesc Psychopharmacol. 2011 Jun;21(3):229-36. doi: 10.1089/cap.2009.0121. PubMed PMID: 21663425.
   4. NCT00337571. Study of Aripiprazole in the Treatment of Children and Adolescents With Autistic Disorder (AD). First Posted: 16^th^ Jun 2006. Accessed: 23^rd^ Jan 2019.
9. McCraken 2002
   1. McCracken JT, McGough J, Shah B, et al. Risperidone in children with autism and serious behavioral problems. N Engl J Med. 2002;347(5):314-21. doi: 10.1056/NEJMoa013171 PMID: 12151468.
   2. Scahill L, McCracken J, McDougle CJ, Aman M, Arnold LE, Tierney E, Cronin P, Davies M, Ghuman J, Gonzalez N, Koenig K, Lindsay R, Martin A, McGough J, Posey DJ, Swiezy N, Volkmar F, Ritz L, Vitiello B. Methodological issues in designing a multisite trial of risperidone in children and adolescents with autism. J Child Adolesc Psychopharmacol. 2001 Winter;11(4):377-88. PubMed PMID: 11838820.
   3. McDougle CJ, Scahill L, McCracken JT, Aman MG, Tierney E, Arnold LE, Freeman BJ, Martin A, McGough JJ, Cronin P, Posey DJ, Riddle MA, Ritz L, Swiezy NB, Vitiello B, Volkmar FR, Votolato NA, Walson P. Research Units on Pediatric Psychopharmacology (RUPP) Autism Network. Background and rationale for an initial controlled study of risperidone. Child Adolesc Psychiatr Clin N Am. 2000 Jan;9(1):201-24. Review. PubMed PMID: 10674197.
   4. Lindsay RL, Eugene Arnold L, Aman MG, Vitiello B, Posey DJ, McDougle CJ, Scahill L, Pachler M, McCracken JT, Tierney E, Bozzolo D. Dietary status and impact of risperidone on nutritional balance in children with autism: a pilot study. J Intellect Dev Disabil. 2006 Dec;31(4):204-9. PubMed PMID: 17178532.
   5. Tobiasova Z, van der Lingen KH, Scahill L, Leckman JF, Zhang Y, Chae W, McCracken JT, McDougle CJ, Vitiello B, Tierney E, Aman MG, Arnold LE, Katsovich L, Hoekstra PJ, Volkmar F, Bothwell AL, Kawikova I. Risperidone-related improvement of irritability in children with autism is not associated with changes in serum of epidermal growth factor and interleukin-13. J Child Adolesc Psychopharmacol. 2011 Dec;21(6):555-64. doi: 10.1089/cap.2010.0134. Epub 2011 Nov 9. PubMed PMID: 22070180; PubMed Central PMCID: PMC3279715.
   6. Levine SZ, Kodesh A, Goldberg Y, Reichenberg A, Furukawa TA, Kolevzon A, Leucht S. Initial severity and efficacy of risperidone in autism: Results from the RUPP trial. Eur Psychiatry. 2016 Feb;32:16-20. doi: 10.1016/j.eurpsy.2015.11.004. Epub 2016 Jan 21. PubMed PMID: 26802979.
   7. Aman MG, Arnold LE, McDougle CJ, Vitiello B, Scahill L, Davies M, McCracken JT, Tierney E, Nash PL, Posey DJ, Chuang S, Martin A, Shah B, Gonzalez NM, Swiezy NB, Ritz L, Koenig K, McGough J, Ghuman JK, Lindsay RL. Acute and long-term safety and tolerability of risperidone in children with autism. J Child Adolesc Psychopharmacol. 2005 Dec;15(6):869-84. PubMed PMID: 16379507.
   8. Williams SK, Scahill L, Vitiello B, Aman MG, Arnold LE, McDougle CJ, McCracken JT, Tierney E, Ritz L, Posey DJ, Swiezy NB, Hollway J, Cronin P, Ghuman J, Wheeler C, Cicchetti D, Sparrow S. Risperidone and adaptive behavior in children with autism. J Am Acad Child Adolesc Psychiatry. 2006 Apr;45(4):431-9. PubMed PMID: 16601648.
   9. NCT00005014. Treatment of Autism in Children and Adolescents. First posted: 3^rd^ Apr 2000. Accessed: 5^th^ Feb 2019.
10. Nagaraj 2006
    1. Nagaraj R, Singhi P, Malhi P. Risperidone in children with autism: randomized, placebo-controlled, double-blind study. J Child Neurol. 2006;21(6):450-5. doi: 10.1177/08830738060210060801. PMID: 16948927.
11. NCT00870727
    1. NCT00870727. Study of Aripiprazole in the Treatment of Pervasive Developmental Disorders. First posted: 27th Mar 2009. Accessed: 18th Feb 2019.
12. NCT01624675
    1. Janssen Research & Development. Synopsis [Protocol RIS-AUT-JPN-01]. A Double-blind, Placebo-controlled Study, Followed by an Open-label Extension Study Evaluating the Efficacy and Safety of Risperidone (R064766) in Children and Adolescents with Irritability Associated with Autistic Disorder. 27^th^ Feb 2015.
    2. NCT01624675. A Study to Evaluate the Efficacy and Safety of Risperidone (R064766) in Children and Adolescents With Irritability Associated With Autistic Disorder. First posted: 21st Jun 2012. Accessed: 18th Feb 2019.
13. Owen 2009
    1. Owen R, Sikich L, Marcus RN, Corey-Lisle P, Manos G, McQuade RD, Carson WH, Findling RL. Aripiprazole in the treatment of irritability in children and adolescents with autistic disorder. Pediatrics. 2009 Dec;124(6):1533-40. doi: 10.1542/peds.2008-3782. PubMed PMID: 19948625.
    2. NCT00332241. Study of Aripiprazole in the Treatment of Children and Adolescents With Autistic Disorder (AD). First Posted: June 1, 2006.
14. Remington 2001
    1. Remington G, Sloman L, Konstantareas M, et al. Clomipramine versus haloperidol in the treatment of autistic disorder: a double-blind, placebo-controlled, crossover study. J Clin Psychopharmacol. 2001 Aug;21(4):440-4. PMID: 11476129.
15. Shea 2004
    1. Shea S, Turgay A, Carroll A, et al. Risperidone in the treatment of disruptive behavioral symptoms in children with autistic and other pervasive developmental disorders. Pediatrics. 2004;114(5):e634-e41. PMID: 15492353.
    2. Pandina GJ, Bossie CA, Youssef E, Zhu Y, Dunbar F. Risperidone improves behavioral symptoms in children with autism in a randomized, double-blind, placebo-controlled trial. J Autism Dev Disord. 2007 Feb;37(2):367-73. PubMed PMID: 17019624.
    3. NCT00261508. A Study of the Effectiveness and Safety of Risperidone Versus Placebo in the Treatment of Children With Autistic Disorder and Other Pervasive Developmental Disorders (PDD). First Posted: 5th Dec 2005. Accessed: 23rd Jan 2019.

## List of excluded studies, with reasons

### Studies comparing two pharmacological interventions, without placebo arm

Ghanizadeh 2014, Lamberti 2016, Malone 2001, Miral 2008, Nicol 2018, NCT01333072, NCT00205699

- Ghanizadeh A, Sahraeizadeh A, Berk M. A head-to-head comparison of aripiprazole and risperidone for safety and treating autistic disorders, a randomized double blind clinical trial. Child Psychiatry Hum Dev. 2014a;45(2):185-92. doi: http://dx.doi.org/10.1007/s10578-013-0390-x. PMID: 23801256
- Lamberti M, Siracusano R, Italiano D, Alosi N, Cucinotta F, Di Rosa G, Germanò E, Spina E, Gagliano A. Head-to-Head Comparison of Aripiprazole and Risperidone in the Treatment of ADHD Symptoms in Children with Autistic Spectrum Disorder and ADHD: A Pilot, Open-Label, Randomized Controlled Study. Paediatr Drugs. 2016 Aug;18(4):319-29. doi: 10.1007/s40272-016-0183-3. PubMed PMID: 27278054.
- Miral S, Gencer O, Inal-Emiroglu FN, et al. Risperidone versus haloperidol in children and adolescents with AD: a randomized, controlled, double-blind trial. Eur Child Adolesc Psychiatry. 2008;17(1):1-8. PMID: 18080171.
- Nicol GE, Yingling MD, Flavin KS, Schweiger JA, Patterson BW, Schechtman KB, Newcomer JW. Metabolic Effects of Antipsychotics on Adiposity and Insulin Sensitivity in Youths: A Randomized Clinical Trial. JAMA Psychiatry. 2018 Aug 1;75(8):788-796. doi: 10.1001/jamapsychiatry.2018.1088. PubMed PMID: 29898210; PubMed Central PMCID: PMC6143095.
- NCT01333072. Biomarkers in Autism of Aripiprazole and Risperidone Treatment (BAART) (BAART). Available at: clinicaltrial.gov; Accessed: 22nd January 2019.
- NCT00205699. Metabolic Effects of Antipsychotics in Children (MEAC). First Posted : 20th Sep 2005. Accessed: 23rd Jan 2019.
- Malone RP, Cater J, Sheikh RM, et al. Olanzapine versus haloperidol in children with autistic disorder: an open pilot study. J Am Acad Child Adolesc Psychiatry. 2001;40(8):887-94. doi:10.1097/00004583-200108000-00009 PMID: 11501687.

### Studies presenting pooled or post-hoc analyses of RCTs

Aman 2008, Aman 2010, Anderson 2007, McDougle 2005, Pandina 2007, Vo 2016, Sanchez 1995, Lewis 2009

- Aman, M. G., Hollway, J. A., McDougle, C. J., Scahill, L., Tierney, E., McCracken, J. T., . . . et al. (2008). Cognitive effects of risperidone in children with autism and irritable behavior. Journal of child and adolescent psychopharmacology, 18(3), 227‐236. doi: 10.1089/cap.2007.0133
- Aman, M. G., Kasper, W., Manos, G., Mathew, S., Marcus, R., Owen, R., & Mankoski, R. (2010). Line-item analysis of the Aberrant Behavior Checklist: results from two studies of aripiprazole in the treatment of irritability associated with autistic disorder. Journal of child and adolescent psychopharmacology, 20(5), 415‐422. doi: 10.1089/cap.2009.0120
- Anderson, G. M., Scahill, L., McCracken, J. T., McDougle, C. J., Aman, M. G., Tierney, E., . . . et al. (2007). Effects of short- and long-term risperidone treatment on prolactin levels in children with autism. Biological psychiatry, 61(4), 545‐550. doi: 10.1016/j.biopsych.2006.02.032
- Lewis, D. W., Couch, D. M., Marcus, R. N., Manos, G., Mankoski, R., & Carson, W. H. (2009). Efficacy and safety of flexibly-dosed aripiprazole for the treatment of irritability associated with autistic disorder in children and adolescents (6–17 years). Annals of neurology, 66(Supp 13 [hardcopy) Suppl 1 [electronic copy]), S110‐111, Abstract no: 143.
- McDougle, C. J., Scahill, L., Aman, M. G., McCracken, J. T., Tierney, E., Davies, M., . . . Vitiello, B. (2005). Risperidone for the core symptom domains of autism: results from the study by the autism network of the research units on pediatric psychopharmacology. The American journal of psychiatry, 162(6), 1142-1148.
- Sanchez, L. E., Adams, P. B., Uysal, S., Hallin, A., Campbell, M., & Small, A. M. (1995). A comparison of live and videotape ratings: clomipramine and haloperidol in autism. Psychopharmacology bulletin, 31(2), 371‐378.
- Vo, L. C., Snyder, C., McCracken, C., McDougle, C. J., McCracken, J. T., Aman, M. GScahill, L. (2016). No Apparent Cardiac Conduction Effects of Acute Treatment with Risperidone in Children with Autism Spectrum Disorder. Journal of child and adolescent psychopharmacology, 26(10), 900-908.

### Studies whose intervention did not meet inclusion criteria

Aman 2015, Arnold 2003, Caicedo 2002, Crosland 2003, Gencer 2008, Kent 2013, Novaes 2008, Sanchez 1996, Perry 1989, NCT00080145, NCT01844700

Aman, M., Rettiganti, M., Nagaraja, H. N., Hollway, J. A., McCracken, J., McDougle, C. J., . . . et al. (2015). Tolerability, Safety, and Benefits of Risperidone in Children and Adolescents with Autism: 21-Month Follow-up After 8-Week Placebo-Controlled Trial. Journal of child and adolescent psychopharmacology, 25(6), 482‐493. doi: 10.1089/cap.2015.0005

Arnold, L. E., Vitiello, B., McDougle, C., Scahill, L., Shah, B., Gonzalez, N. M., . . . Tierney, E. (2003). Parent-defined target symptoms respond to risperidone in RUPP autism study: customer approach to clinical trials. Journal of the american academy of child and adolescent psychiatry, 42(12), 1443-1450.

Caicedo, C., & Williams, S. H. (2002). Risperidone improves behavior in children with autism. Journal of family practice, 51(11), 915.

Crosland, K. A., Zarcone, J. R., Lindauer, S. E., Valdovinos, M. G., Zarcone, T. J., Hellings, J. A., & Schroeder, S. R. (2003). Use of functional analysis methodology in the evaluation of medication effects. Journal of autism and developmental disorders, 33(3), 271‐279.

Gencer, O., Emiroglu, F. N. I., Miral, S., Baykara, B., Baykara, A., & Dirik, E. (2008). Comparison of long-term efficacy and safety of risperidone and haloperidol in children and adolescents with autistic disorder. An open label maintenance study. European child & adolescent psychiatry, 17(4), 217-225.

Kent, J. M., Hough, D., Singh, J., Karcher, K., & Pandina, G. (2013). An open-label extension study of the safety and efficacy of risperidone in children and adolescents with autistic disorder. Journal of child and adolescent psychopharmacology, 23(10), 676‐686. doi: 10.1089/cap.2012.0058

Novaes, C. M., Ponde, M. P., & Freire, A. C. C. (2008). Control of psychomotor agitation and aggressive behavior in patients with autistic disorder: a retrospective chart review. Arquivos de neuro-psiquiatria, 66(3B), 646-651.

Perry R, Campbell M, Adams P, et al. Long-term efficacy of haloperidol in autistic children: continuous versus discontinuous drug administration. J Am Acad Child Adolesc Psychiatry. 1989;28(1):87-92. doi:10.1097/00004583-198901000-00016 PMID: 2914841.

Sanchez, L. E., Campbell, M., Small, A. M., Cueva, J. E., Armenteros, J. L., & Adams, P. B. (1996). A pilot study of clomipramine in young autistic children. Journal of the american academy of child and adolescent psychiatry, 35(4), 537-544. doi: 10.1097/00004583-199604000-00021

NCT00080145. RUPP PI PDD: Drug and Behavioral Therapy for Children With Pervasive Developmental Disorders. Available at: clinicaltrial.gov; Accessed: 9th Jan 2019.

NCT01844700. 1/2-MC4R Genotype and Pediatric Antipsychotic Drug- Induced Weight Gain. Available at: clinicaltrial.gov; Accessed: 18th Dec 2019.

### Studies for which could not find the full-text

Elizur 1975, Miller 1979

Elizur A, Davidson S. The evaluation of the anti-autistic activity of sulpiride. Curr Ther Res Clin Exp. 1975 Oct;18(4):578-84. PubMed PMID: 810319.

Miller B, Wallis H. [Mode of action of sulpiride in autistic children. A double blind study]. MMW Munch Med Wochenschr. 1979 May 11;121(19):667-9. German. PubMed PMID: 112394.

### Studies whose population did not meet inclusion criteria (as it included only adults with ASD)

McDougle 1998

McDougle CJ, Holmes JP, Carlson DC, Pelton GH, Cohen DJ, Price LH. A double-blind, placebo-controlled study of risperidone in adults with autistic disorder and other pervasive developmental disorders. Arch Gen Psychiatry. 1998 Jul;55(7):633-41. PubMed PMID: 9672054.

### Studies whose design did not meet inclusion criteria

NCT00166595, NCT00691080, NCT00619190

NCT00166595. Pharmacogenetics of Risperidone in Children With Pervasive Developmental Disorder (PDD). Available at: clinicaltrial.gov; Accessed: 18th Dec 2019.

NCT00691080. Understanding Sleep Problems in Children With Autism Spectrum Disorder (REST). Available at: clinicaltrial.gov; Accessed: 18th Dec 2019.

NCT00619190. Study of Aripiprazole to Treat Children and Adolescents With Autism (PAIRS). Available at: clinicaltrial.gov; Accessed: 18th Dec 2019.

### Studies that did not report any result (awaiting assessment)

- NCT01171937. Risperidone Treatment In Children With Autism Spectrum Disorder And High Levels Of Repetitive Behavior (ProjectV). First Posted: 29th Jul 2010. Accessed: 23rd Jan 2019.
- NCT00147394. Risperidone Pharmacokinetics in Children With Pervasive Developmental Disorder (PDD). First Posted: 7th Sep 2005. Accessed: 23rd Jan 2019.
- NCT00198107. Evaluating the Effectiveness of Aripiprazole and D-Cycloserine to Treat Symptoms Associated With Autism. Available at: clinicaltrial.gov; Accessed: 18th Dec 2019. Results published on March 29, 2019, after the completion of our systematic review for the ASD guidelines development purpose.
- NCT00468130. Efficacy of Aripiprazole Versus Placebo in the Reduction of Aggressive and Aberrant Behavior in Autistic Children (Abilify). Available at: clinicaltrial.gov; Accessed: 18th Dec 2019.
- NCT00057408. A Controlled Study of Olanzapine in Children With Autism. Available at: clinicaltrial.gov; Accessed: 18th Dec 2019.

### Augmentation trials with no placebo arm

- Akhondzadeh, S., & Asadabadi, M. (2012). Risperidone plus celecoxib in children with autistic disorder: a double-blind, randomized trial. British journal of clinical pharmacology, 73(6), 983‐984. doi: 10.1111/j.1365-2125.2012.04253.x
- Akhondzadeh, S., Fallah, J., Mohammadi, M. R., Imani, R., Mohammadi, M., Salehi, B., . . . et al. (2010). Double-blind placebo-controlled trial of pentoxifylline added to risperidone: effects on aberrant behavior in children with autism. Progress in neuro-psychopharmacology & biological psychiatry, 34(1), 32‐36. doi: 10.1016/j.pnpbp.2009.09.012
- Akhondzadeh, S., Tajdar, H., Mohammadi, M. R., Mohammadi, M., Nouroozinejad, G. H., Shabstari, O. L., & Ghelichnia, H. A. (2008). A double-blind placebo controlled trial of piracetam added to risperidone in patients with autistic disorder. Child psychiatry and human development, 39(3), 237‐245. doi: 10.1007/s10578-007-0084-3
- Asadabadi, M., Mohammadi, M.-R., Ghanizadeh, A., Modabbernia, A., Ashrafi, M., Hassanzadeh, E., . . . Akhondzadeh, S. (2013). Celecoxib as adjunctive treatment to risperidone in children with autistic disorder: a randomized, double-blind, placebo-controlled trial. Psychopharmacology, 225(1), 51-59. doi: https://dx.doi.org/10.1007/s00213-012-2796-8
- Ghaleiha, A., Alikhani, R., Kazemi, M.-R., Mohammadi, M.-R., Mohammadinejad, P., Zeinoddini, A., . . . Akhondzadeh, S. (2016). Minocycline as Adjunctive Treatment to Risperidone in Children with Autistic Disorder: A Randomized, Double-Blind Placebo-Controlled Trial. Journal of child and adolescent psychopharmacology, 26(9), 784-791.
- Ghaleiha, A., Asadabadi, M., Mohammadi, M.-R., Shahei, M., Tabrizi, M., Hajiaghaee, R., . . . Akhondzadeh, S. (2013). Memantine as adjunctive treatment to risperidone in children with autistic disorder: a randomized, double-blind, placebo-controlled trial. The international journal of neuropsychopharmacology, 16(4), 783-789. doi: https://dx.doi.org/10.1017/S1461145712000880
- Ghaleiha, A., Ghyasvand, M., Mohammadi, M.-R., Farokhnia, M., Yadegari, N., Tabrizi, M., . . . Akhondzadeh, S. (2014). Galantamine efficacy and tolerability as an augmentative therapy in autistic children: A randomized, double-blind, placebo-controlled trial. Journal of psychopharmacology (oxford, england), 28(7), 677-685. doi: https://dx.doi.org/10.1177/0269881113508830
- Ghaleiha, A., Mohammadi, E., Mohammadi, M.-R., Farokhnia, M., Modabbernia, A., Yekehtaz, H., . . . Akhondzadeh, S. (2013). Riluzole as an adjunctive therapy to risperidone for the treatment of irritability in children with autistic disorder: a double-blind, placebo-controlled, randomized trial. Paediatric drugs, 15(6), 505-514. doi: https://dx.doi.org/10.1007/s40272-013-0036-2
- Ghaleiha, A., Rasa, S. M., Nikoo, M., Farokhnia, M., Mohammadi, M.-R., & Akhondzadeh, S. (2015). A pilot double-blind placebo-controlled trial of pioglitazone as adjunctive treatment to risperidone: Effects on aberrant behavior in children with autism. Psychiatry research, 229(1-2), 181-187. doi: https://dx.doi.org/10.1016/j.psychres.2015.07.043
- Ghanizadeh, A., & Ayoobzadehshirazi, A. (2015). A randomized double-blind placebo-controlled clinical trial of adjuvant buspirone for irritability in autism. Pediatric neurology, 52(1), 77‐81. doi: 10.1016/j.pediatrneurol.2014.09.017
- Ghanizadeh, A., & Moghimi-Sarani, E. (2013). A randomized double blind placebo controlled clinical trial of N-Acetylcysteine added to risperidone for treating autistic disorders. BMC psychiatry, 13, 196. doi: 10.1186/1471-244X-13-196
- Hajizadeh-Zaker, R., Ghajar, A., Mesgarpour, B., Afarideh, M., Mohammadi, M.-R., & Akhondzadeh, S. (2018). l-Carnosine As an Adjunctive Therapy to Risperidone in Children with Autistic Disorder: A Randomized, Double-Blind, Placebo-Controlled Trial. Journal of child and adolescent psychopharmacology, 28(1), 74-81. doi: https://dx.doi.org/10.1089/cap.2017.0026
- Hasanzadeh, E., Mohammadi, M.-R., Ghanizadeh, A., Rezazadeh, S.-A., Tabrizi, M., Rezaei, F., & Akhondzadeh, S. (2012). A double-blind placebo controlled trial of Ginkgo biloba added to risperidone in patients with autistic disorders. Child psychiatry and human development, 43(5), 674-682. doi: https://dx.doi.org/10.1007/s10578-012-0292-3
- Moazen-Zadeh, E., Shirzad, F., Karkhaneh-Yousefi, M. A., Khezri, R., Mohammadi, M. R., & Akhondzadeh, S. (2018). Simvastatin as an Adjunctive Therapy to Risperidone in Treatment of Autism: a Randomized, Double-Blind, Placebo-Controlled Clinical Trial. Journal of child and adolescent psychopharmacology, 28(1), 82‐89. doi: 10.1089/cap.2017.0055
- Mohammadi, M.-R., Yadegari, N., Hassanzadeh, E., Farokhnia, M., Yekehtaz, H., Mirshafiee, O., & Akhondzadeh, S. (2013). Double-blind, placebo-controlled trial of risperidone plus amantadine in children with autism: a 10-week randomized study. Clinical neuropharmacology, 36(6), 179-184. doi: https://dx.doi.org/10.1097/WNF.0b013e3182a9339d
- Moharreri, F., Abdollahian, E., Hosseini, S. A., & Mirzadeh, M. (2017). Comparative Study on the Effect of Risperidone and its Combination with Naltrexone in Pediatric Patients with Autistic Spectrum Disorders: A Clinical Trial Study. International Journal of Pediatrics-Mashhad, 5(12), 6375-6382. doi: 10.22038/ijp.2017.18557.1516
- Nikoo, M., Radnia, H., Farokhnia, M., Mohammadi, M.-R., & Akhondzadeh, S. (2015). N-acetylcysteine as an adjunctive therapy to risperidone for treatment of irritability in autism: a randomized, double-blind, placebo-controlled clinical trial of efficacy and safety. Clinical neuropharmacology, 38(1), 11-17. doi: https://dx.doi.org/10.1097/WNF.0000000000000063
- Rezaei, M., Moradi, A., Tehrani-Doost, M., Hassanabadi, H., & Khosroabadi, R. (2018). Effects of Combining Medication and Pivotal Response Treatment on Aberrant Behavior in Children with Autism Spectrum Disorder. Children (Basel, Switzerland), 5(2). doi: https://dx.doi.org/10.3390/children5020019
- Rezaei, V., Mohammadi, M.-R., Ghanizadeh, A., Sahraian, A., Tabrizi, M., Rezazadeh, S.-A., & Akhondzadeh, S. (2010). Double-blind, placebo-controlled trial of risperidone plus topiramate in children with autistic disorder. Progress in neuro-psychopharmacology & biological psychiatry, 34(7), 1269-1272. doi: https://dx.doi.org/10.1016/j.pnpbp.2010.07.005
- Scahill, L., Aman, M. G., McDougle, C. J., Arnold, L. E., McCracken, J. T., Handen, B., . . . Vitiello, B. (2009). Trial design challenges when combining medication and parent training in children with pervasive developmental disorders. Journal of autism and developmental disorders, 39(5), 720-729. doi: https://dx.doi.org/10.1007/s10803-008-0675-2
- Scahill, L., McDougle, C. J., Aman, M. G., Johnson, C., Handen, B., Bearss, K., . . . Research Units on Pediatric Psychopharmacology Autism, N. (2012). Effects of risperidone and parent training on adaptive functioning in children with pervasive developmental disorders and serious behavioral problems. Journal of the american academy of child and adolescent psychiatry, 51(2), 136-146. doi: <https://dx.doi.org/10.1016/j.jaac.2011.11.010>
- Calarge CA, Ziegler EE, Del Castillo N, Aman M, McDougle CJ, Scahill L, McCracken JT, Arnold LE. Iron homeostasis during risperidone treatment in children and adolescents. J Clin Psychiatry. 2015 Nov;76(11):1500-5. doi: 10.4088/JCP.14m09258. PubMed PMID: 26301448.

### Studies that did not report any data of interest

- Research Units on Pediatric Psychopharmacology (RUPP). Risperidone treatment of autistic disorder: longer-term benefits and blinded discontinuation after 6 months. Am J Psychiatry. 2005;162(7):1361-9.
- Troost PW, Lahuis BE, Steenhuis MP, et al. Long-term effects of risperidone in children with autism spectrum disorders: a placebo discontinuation study. J Am Acad Child Adolesc Psychiatry. 2005;44(11):1137-44. doi:10.1097/01.chi.0000177055.11229.76 PMID: 16239862.
- Troost PW, Althaus M, Lahuis BE, Buitelaar JK, Minderaa RB, Hoekstra PJ. Neuropsychological effects of risperidone in children with pervasive developmental disorders: a blinded discontinuation study. J Child Adolesc Psychopharmacol. 2006 Oct;16(5):561-73. PubMed PMID: 17069545.
- Anderson LT, Campbell M, Grega DM, Perry R, Small AM, Green WH. Haloperidol in the treatment of infantile autism: effects on learning and behavioral symptoms. Am J Psychiatry. 1984 Oct;141(10):1195-202. PubMed PMID: 6385731.
- Campbell M, Anderson LT, Small AM, Perry R, Green WH, Caplan R. The effects of haloperidol on learning and behavior in autistic children. J Autism Dev Disord. 1982 Jun;12(2):167-75. PubMed PMID: 7174605.
- Anderson LT, Campbell M, Adams P, et al. The effects of haloperidol on discrimination learning and behavioral symptoms in autistic children. J Autism Dev Disord. 1989 Jun;19(2):227-39. PMID: 2663834.
- Cohen IL, Campbell M, Posner D. A study of haloperidol in young autistic children: a within-subjects design using objective rating scales. Psychopharmacol Bull. 1980 Jul;16(3):63-5. PubMed PMID: 7403407.
- Cohen IL, Campbell M, Posner D, Small AM, Triebel D, Anderson LT. Behavioral effects of haloperidol in young autistic children. An objective analysis using a within-subjects reversal design. J Am Acad Child Psychiatry. 1980 Autumn;19(4):665-77. PubMed PMID: 7204797.
- Hellings JA, Zarcone JR, Reese RM, Valdovinos MG, Marquis JG, Fleming KK, Schroeder SR. A crossover study of risperidone in children, adolescents and adults with mental retardation. J Autism Dev Disord. 2006 Apr;36(3):401-11. PubMed PMID: 16596465.
- Hellings JA, Zarcone JR, Crandall K, Wallace D, Schroeder SR. Weight gain in a controlled study of risperidone in children, adolescents and adults with mental retardation and autism. J Child Adolesc Psychopharmacol. 2001 Fall;11(3):229-38. PubMed PMID: 11642473.
- Hellings JA, Zarcone JR, Valdovinos MG, Reese RM, Gaughan E, Schroeder SR. Risperidone-induced prolactin elevation in a prospective study of children, adolescents, and adults with mental retardation and pervasive developmental disorders. J Child Adolesc Psychopharmacol. 2005 Dec;15(6):885-92. PubMed PMID: 16379508.

# Full References for included and excluded studies from the search strategy for observational studies.

## Full References for included observational studies

1. Bowker A, D'Angelo NM, Hicks R, Wells K. Treatments for autism: parental choices and perceptions of change. J Autism Dev Disord. 2011 Oct;41(10):1373-82. doi: 10.1007/s10803-010-1164-y. PubMed PMID: 21161676.
2. Downs J, Hotopf M, Ford T, Simonoff E, Jackson RG, Shetty H, Stewart R, Hayes RD. Clinical predictors of antipsychotic use in children and adolescents with autism spectrum disorders: a historical open cohort study using electronic health records. Eur Child Adolesc Psychiatry. 2016 Jun;25(6):649-58. doi: 10.1007/s00787-015-0780-7. Epub 2015 Oct 15. PubMed PMID: 26472118; PubMed Central PMCID: PMC4889626.
3. Jobski K, Höfer J, Hoffmann F, Bachmann C. Use of psychotropic drugs in patients with autism spectrum disorders: a systematic review. Acta Psychiatr Scand. 2017 Jan;135(1):8-28. doi: 10.1111/acps.12644. Epub 2016 Sep 13. Review. PubMed PMID: 27624381.
4. Ramerman L, Hoekstra PJ, de Kuijper G. Exploring barriers and facilitators in the implementation and use of guideline recommendations on antipsychotic drug prescriptions for people with intellectual disability. J Appl Res Intellect Disabil. 2018 Nov;31(6):1062-1070. doi: 10.1111/jar.12461. Epub 2018 Jun 20. PubMed PMID: 29923275.
5. Rodday AM, Parsons SK, Mankiw C, Correll CU, Robb AS, Zima BT, Saunders TS, Leslie LK. Child and adolescent psychiatrists' reported monitoring behaviors for second-generation antipsychotics. J Child Adolesc Psychopharmacol. 2015 May;25(4):351-61. doi: 10.1089/cap.2014.0156. Epub 2015 Apr 28. PubMed PMID: 25918843; PubMed Central PMCID: PMC4442598.
6. Ronsley R, Raghuram K, Davidson J, Panagiotopoulos C. Barriers and facilitators to implementation of a metabolic monitoring protocol in hospital and community settings for second-generation antipsychotic-treated youth. J Can Acad Child Adolesc Psychiatry. 2011 May;20(2):134-41. PubMed PMID: 21541103; PubMed Central PMCID: PMC3085673
7. Tierney E, Aman M, Stout D, Pappas K, Arnold LE, Vitiello B, Scahill L, McDougle C, McCracken J, Wheeler C, Martin A, Posey D, Shah B. Parent satisfaction in a multi-site acute trial of risperidone in children with autism: a social validity study. Psychopharmacology (Berl). 2007 Mar;191(1):149-57. Epub 2006 Nov 23. PubMed PMID: 17123125.

## List of excluded studies, with reasons

### Studies that did not report any data of interest

1. Arnold LE, Farmer C, Kraemer HC, Davies M, Witwer A, Chuang S, DiSilvestro R, McDougle CJ, McCracken J, Vitiello B, Aman MG, Scahill L, Posey DJ, Swiezy NB. Moderators, mediators, and other predictors of risperidone response in children with autistic disorder and irritability. J Child Adolesc Psychopharmacol. 2010 Apr;20(2):83-93. doi: 10.1089/cap.2009.0022. PubMed PMID: 20415603; PubMed Central PMCID: PMC2865212.
2. Arnold LE, Vitiello B, McDougle C, Scahill L, Shah B, Gonzalez NM, Chuang S, Davies M, Hollway J, Aman MG, Cronin P, Koenig K, Kohn AE, McMahon DJ, Tierney E. Parent-defined target symptoms respond to risperidone in RUPP autism study: customer approach to clinical trials. J Am Acad Child Adolesc Psychiatry. 2003 Dec;42(12):1443-50. PubMed PMID: 14627879.
3. Lindsay RL, Eugene Arnold L, Aman MG, Vitiello B, Posey DJ, McDougle CJ, Scahill L, Pachler M, McCracken JT, Tierney E, Bozzolo D. Dietary status and impact of risperidone on nutritional balance in children with autism: a pilot study. J Intellect Dev Disabil. 2006 Dec;31(4):204-9. PubMed PMID: 17178532.
4. Panagiotopoulos C, Ronsley R, Elbe D, Davidson J, Smith DH. First do no harm: promoting an evidence-based approach to atypical antipsychotic use in children and adolescents. J Can Acad Child Adolesc Psychiatry. 2010 May;19(2):124-37. PubMed PMID: 20467549; PubMed Central PMCID: PMC2868560.
5. Vitiello B, Aman MG, Scahill L, McCracken JT, McDougle CJ, Tierney E, Davies M, Arnold LE. Research knowledge among parents of children participating in a randomized clinical trial. J Am Acad Child Adolesc Psychiatry. 2005 Feb;44(2):145-9. PubMed PMID: 15689727.

### Studies whose population did not meet inclusion criteria

1. Boucher D, Gascon H, Sirois C, Garant M.2015. The use of psychotropic medications in autism spectrum disorder: The parents' perspective. Pharmacoepidemiology and Drug Safety. 2015, 24(SUPPL. 1): 284-285.
2. Guler AS, Yazgan Y, Pelin AU. Autistic traits and factors related to a clinical decision to use risperidone in children with Attention Deficit Hyperactivity Disorder. Klinik Psikofarmakoloji Bulteni / Bulletin of Clinical Psychopharmacology. 2014,24(4):333-341.

### Studies for which could not find the full-text

1. Bonnot O, Holzer L. [Use of antipsychotics in child and adolescent]. Neuropsychiatrie de l'Enfance et de l'Adolescence. 2012; 60(1):12-9.
