# Supplementary material for: Acceptability, equity, and feasibility of using antipsychotics in children and adolescents with autism spectrum disorder: a systematic review
Source: BMC Psychiatry. 2020 Nov 25;20:561. doi: 10.1186/s12888-020-02956-8 (PMC7687819; doi:10.1186/s12888-020-02956-8)
Supplement: Supplementary file 4 — Additional file 4. Risk of bias summary. [file 12888_2020_2956_MOESM4_ESM.docx]

**Additional file 4. Risk of Bias Summary.**

**These are review authors' judgments about each risk of bias item for each included study.**

| **Study** | **Random sequence generation**  **(selection bias)** | **Allocation concealment (selection bias)** | **Blinding of participants and personnel (performance bias)** | **Blinding of outcome assessment**  **(detection bias)** | **Incomplete outcome data**  **(attrition bias)** | **Selective reporting (reporting bias)** |
| --- | --- | --- | --- | --- | --- | --- |
| Campbell, 1978 | 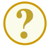 | 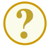 | 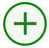 | 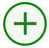 | 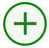 | 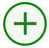 |
| Findling, 2014 | 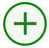 | 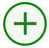 | 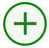 | 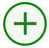 | **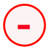** | 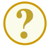 |
| Hollander, 2006 | 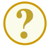 | 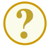 | 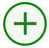 | 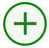 | 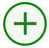 | 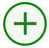 |
| Ichikawa, 2017 | 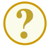 | 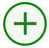 | 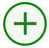 | 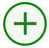 | 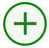 | 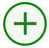 |
| Kent, 2013 | 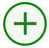 | 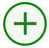 | 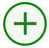 | 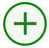 | 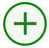 | 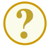 |
| Loebel, 2016 | 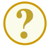 | 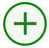 | 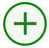 | 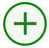 | 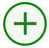 | 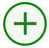 |
| Luby, 2006 | 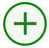 | **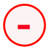** | 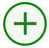 | 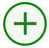 | 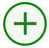 | **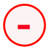** |
| Marcus, 2009 | 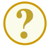 | 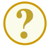 | 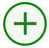 | 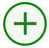 | 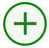 | 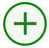 |
| McCraken, 2002 | 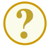 | 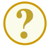 | 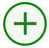 | 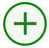 | **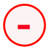** | 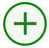 |
| Nagaraj, 2006 | 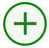 | 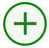 | 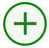 | 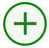 | 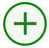 | 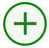 |
| NCT00870727 | 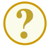 | 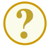 | 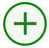 | 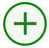 | 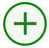 | 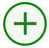 |
| NCT01624675 | 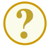 | 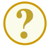 | 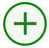 | 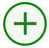 | **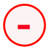** | 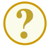 |
| Owen, 2009 | 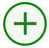 | 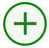 | 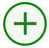 | 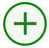 | 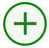 | 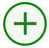 |
| Remington, 2001 | 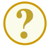 | 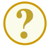 | 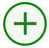 | 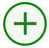 | **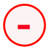** | 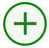 |
| Shea, 2004 | 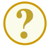 | 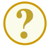 | 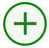 | 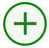 | 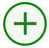 | 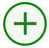 |

Abbreviations:
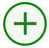
 = Low risk of bias;
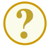
 = Unclear risk of bias; **
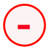
** = High risk of bias.

**References**

- Campbell M, Anderson LT, Meier M, Cohen IL, Small AM, Samit C, Sachar EJ. A comparison of haloperidol and behavior therapy and their interaction in autistic children. J Am Acad Child Psychiatry. 1978 Autumn;17(4):640-55. PubMed PMID: 370186.
- Findling RL, Mankoski R, Timko K, Lears K, McCartney T, McQuade RD, Eudicone JM, Amatniek J, Marcus RN, Sheehan JJ. A randomized controlled trial investigating the safety and efficacy of aripiprazole in the long-term maintenance treatment of pediatric patients with irritability associated with autistic disorder. J Clin Psychiatry. 2014 Jan;75(1):22-30. doi:10.4088/JCP.13m08500. PubMed PMID: 24502859.
- Hollander E, Wasserman S, Swanson EN, et al. A double-blind placebo-controlled pilot study of olanzapine in childhood/adolescent pervasive developmental disorder. J Child Adolesc Psychopharmacol. 2006;16(5):541-8. doi:10.1089/cap.2006.16.541. PMID: 17069543.
- Ichikawa H, Mikami K, Okada T, Yamashita Y, Ishizaki Y, Tomoda A, Ono H, Usuki C, Tadori Y. Aripiprazole in the Treatment of Irritability in Children and Adolescents with Autism Spectrum Disorder in Japan: A Randomized, Double-blind, Placebo-controlled Study. Child Psychiatry Hum Dev. 2017 Oct;48(5):796-806. doi: 10.1007/s10578-016-0704-x. PubMed PMID: 28004215; PubMed Central PMCID: PMC5617873.
- Kent JM, Kushner S, Ning X, et al. Risperidone dosing in children and adolescents with autistic disorder: a double-blind, placebo-controlled study. J Autism Dev 197 Disord. 2013 Aug;43(8):1773-83. doi: 10.1007/s10803-012-1723-5. PMID: 23212807.
- Loebel A, Brams M, Goldman RS, et al. Lurasidone for the treatment of irritability with autistic disorder. J Autism Dev Disord. 2016;46:1153-63.
- Luby J, Mrakotsky C, Stalets MM, et al. Risperidone in preschool children with autistic spectrum disorders: an investigation of safety and efficacy. J Child Adoles Psychopharmacol. 2006;16(5):575-87. doi:10.1089/cap.2006.16.575. PMID: 17069546.
- Marcus RN, Owen R, Kamen L, Manos G, McQuade RD, Carson WH, Aman MG. A placebo-controlled, fixed-dose study of aripiprazole in children and adolescents with irritability associated with autistic disorder. J Am Acad Child Adolesc Psychiatry. 2009 Nov;48(11):1110-9. doi: 10.1097/CHI.0b013e3181b76658. PubMed PMID: 19797985.
- McCracken JT, McGough J, Shah B, et al. Risperidone in children with autism and serious behavioral problems. N Engl J Med. 2002;347(5):314-21. doi: 10.1056/NEJMoa013171 PMID: 12151468.
- Nagaraj R, Singhi P, Malhi P. Risperidone in children with autism: randomized, placebo-controlled, double-blind study. J Child Neurol. 2006;21(6):450-5. doi: 10.1177/08830738060210060801. PMID: 16948927.
- NCT00870727. Study of Aripiprazole in the Treatment of Pervasive Developmental Disorders. First posted: 27^th^ Mar 2009. Accessed: 18^th^ Feb 2019.
- NCT01624675. A Study to Evaluate the Efficacy and Safety of Risperidone (R064766) in Children and Adolescents With Irritability Associated With Autistic Disorder. First posted: 21^st^ Jun 2012. Accessed: 18^th^ Feb 2019.
- Owen R, Sikich L, Marcus RN, Corey-Lisle P, Manos G, McQuade RD, Carson WH, Findling RL. Aripiprazole in the treatment of irritability in children and adolescents with autistic disorder. Pediatrics. 2009 Dec;124(6):1533-40. doi: 10.1542/peds.2008-3782. PubMed PMID: 19948625.
- Remington G, Sloman L, Konstantareas M, et al. Clomipramine versus haloperidol in the treatment of autistic disorder: a double-blind, placebo-controlled, crossover study. J Clin Psychopharmacol. 2001 Aug;21(4):440-4. PMID: 11476129.
- Shea S, Turgay A, Carroll A, et al. Risperidone in the treatment of disruptive behavioral symptoms in children with autistic and other pervasive developmental disorders. Pediatrics. 2004;114(5):e634-e41. PMID: 15492353.
