# Supplementary material for: Acceptability, equity, and feasibility of using antipsychotics in children and adolescents with autism spectrum disorder: a systematic review
Source: BMC Psychiatry. 2020 Nov 25;20:561. doi: 10.1186/s12888-020-02956-8 (PMC7687819; doi:10.1186/s12888-020-02956-8)
Supplement: Supplementary file 5 — Additional file 5. Newcastle Ottawa Scale (NOS). [file 12888_2020_2956_MOESM5_ESM.docx]

| **Additional file 5. Newcastle Ottawa Scale (NOS) for observational studies** | | | | | | | |
| --- | --- | --- | --- | --- | --- | --- | --- |
| Cross-sectional studies ^1^ | | | | | | | |
|  | Selection | | | | Comparability | Outcome | |
| Study | Representativeness of the sample | Sample size | Non-respondents | Ascertainment of the exposure (risk factor) (maximum 2 stars) | Comparability of outcome groups (maximum 2 stars) | Assessment of the outcome (maximum 2 stars) | Statistical test |
| *Bowker 2011* | * | * | - | - | - | * | * |
| *Ramerman 2018* | - | - | - | - | - | - | - |
| *Rodday 2015* | * | * | - | * | ** | * | * |
| *Ronsley 2011* | * | - | - | * | - | * | - |
| *Tierney 2007* | * | * | * | ** | ** | * | * |
| Cohort studies | | | | | | | |
|  | Selection | | | | Comparability | Outcome | |
| Study | Representativeness of the exposed cohort | Selection of the non-exposed cohort | Ascertainment of exposure | Demonstration that outcome of interest was not present at start of study | Comparability of cohorts (maximum 2 stars) | Assessment of outcome | Adequacy of follow up (maximum 2 stars) |
| *Downs 2016* | * | * | * | * | ** | * | ** |

^1^ Quality assessment of cross-sectional included studies performed through Newcastle - Ottawa Quality Assessment Scale adapted for cross sectional studies (Modesti 2016)

**References**

1. Bowker A, D'Angelo NM, Hicks R, Wells K. Treatments for autism: parental choices and perceptions of change. J Autism Dev Disord. 2011 Oct;41(10):1373-82. doi: 10.1007/s10803-010-1164-y. PubMed PMID: 21161676.
2. Tierney E, Aman M, Stout D, Pappas K, Arnold LE, Vitiello B, Scahill L, McDougle C, McCracken J, Wheeler C, Martin A, Posey D, Shah B. Parent satisfaction in a multi-site acute trial of risperidone in children with autism: a social validity study. Psychopharmacology (Berl). 2007 Mar;191(1):149-57. Epub 2006 Nov 23. PubMed PMID: 17123125.
3. Downs J, Hotopf M, Ford T, Simonoff E, Jackson RG, Shetty H, Stewart R, Hayes RD. Clinical predictors of antipsychotic use in children and adolescents with autism spectrum disorders: a historical open cohort study using electronic health records. Eur Child Adolesc Psychiatry. 2016 Jun;25(6):649-58. doi: 10.1007/s00787-015-0780-7. Epub 2015 Oct 15. PubMed PMID: 26472118; PubMed Central PMCID: PMC4889626.
4. Ramerman L, Hoekstra PJ, de Kuijper G. Exploring barriers and facilitators in the implementation and use of guideline recommendations on antipsychotic drug prescriptions for people with intellectual disability. J Appl Res Intellect Disabil. 2018 Nov;31(6):1062-1070. doi: 10.1111/jar.12461. Epub 2018 Jun 20. PubMed PMID: 29923275.
5. Rodday AM, Parsons SK, Mankiw C, Correll CU, Robb AS, Zima BT, Saunders TS, Leslie LK. Child and adolescent psychiatrists' reported monitoring behaviors for second-generation antipsychotics. J Child Adolesc Psychopharmacol. 2015 May;25(4):351-61. doi: 10.1089/cap.2014.0156. Epub 2015 Apr 28. PubMed PMID: 25918843; PubMed Central PMCID: PMC4442598.
6. Ronsley R, Raghuram K, Davidson J, Panagiotopoulos C. Barriers and facilitators to implementation of a metabolic monitoring protocol in hospital and community settings for second-generation antipsychotic-treated youth. J Can Acad Child Adolesc Psychiatry. 2011 May;20(2):134-41. PubMed PMID: 21541103; PubMed Central PMCID: PMC3085673
