# Supplementary material for: Acceptability, equity, and feasibility of using antipsychotics in children and adolescents with autism spectrum disorder: a systematic review
Source: BMC Psychiatry. 2020 Nov 25;20:561. doi: 10.1186/s12888-020-02956-8 (PMC7687819; doi:10.1186/s12888-020-02956-8)
Supplement: Supplementary file 6 — Additional file 6. AMSTAR 2. [file 12888_2020_2956_MOESM6_ESM.docx]

Additional file 6.

## Methodological quality assessment of Systematic Reviews through AMSTAR 2 critical appraisal tool

|  |  | Item | | | | | | | | | | | | | | | | | Overall rating |
| --- | --- | --- | --- | --- | --- | --- | --- | --- | --- | --- | --- | --- | --- | --- | --- | --- | --- | --- | --- |
| Study | 1 | 2 | 3 | 4 | 5 | 6 | 7 | 8 | 9 RCT | 9 NRSI | 10 | 11 RCT | 11 NRSI | 12 | 13 | 14 | 15 | 16 |  |
| Jobski 2017 | Yes | No | NA | Partial Yes | Yes | Yes | No | Yes | NA | NA | No | No MA | No MA | No MA | No | Yes | No MA | Yes | **Low** |

**Legend:** ***MA****: Meta-analysis;* ***NA****: Not Applicable.*

## List of items assessed by AMSTAR 2 (Shea 2017)

1. Did the research questions and inclusion criteria for the review include the components of PICO?

2. Did the report of the review contain an explicit statement that the review methods were established prior to the conduct of the review and did the report justify any significant deviations from the protocol?

3. Did the review authors explain their selection of the study designs for inclusion in the review?

4. Did the review authors use a comprehensive literature search strategy?

5. Did the review authors perform study selection in duplicate?

6. Did the review authors perform data extraction in duplicate?

7. Did the review authors provide a list of excluded studies and justify the exclusions?

8. Did the review authors describe the included studies in adequate detail?

9. Did the review authors use a satisfactory technique for assessing the risk of bias (RoB) in individual studies that were included in the review?

10. Did the review authors report on the sources of funding for the studies included in the review?

11. If meta-analysis was performed did the review authors use appropriate methods for statistical combination of results?

12. If meta-analysis was performed, did the review authors assess the potential impact of RoB in individual studies on the results of the meta-analysis or other evidence synthesis?

13. Did the review authors account for RoB in individual studies when interpreting/ discussing the results of the review?

14. Did the review authors provide a satisfactory explanation for, and discussion of, any heterogeneity observed in the results of the review?

15. If they performed quantitative synthesis did the review authors carry out an adequate investigation of publication bias (small study bias) and discuss its likely impact on the results of the review?

16. Did the review authors report any potential sources of conflict of interest, including any funding they received for conducting the review?

## References:

- Jobski K, Höfer J, Hoffmann F, Bachmann C. Use of psychotropic drugs in patients with autism spectrum disorders: a systematic review. Acta Psychiatr Scand. 2017 Jan;135(1):8-28. doi: 10.1111/acps.12644. Epub 2016 Sep 13. Review. PubMed PMID: 27624381.
- Shea BJ, Reeves BC, Wells G, Thuku M, Hamel C, Moran J, Moher D, Tugwell P, Welch V, Kristjansson E, Henry DA. AMSTAR 2: a critical appraisal tool for systematic reviews that include randomised or non-randomised studies of healthcare interventions, or both. BMJ. 2017 Sep 21;358:j4008. doi: 10.1136/bmj.j4008. PubMed PMID: 28935701; PubMed Central PMCID: PMC5833365
