# Supplementary material for: Acceptability, equity, and feasibility of using antipsychotics in children and adolescents with autism spectrum disorder: a systematic review
Source: BMC Psychiatry. 2020 Nov 25;20:561. doi: 10.1186/s12888-020-02956-8 (PMC7687819; doi:10.1186/s12888-020-02956-8)

Additional file 7: Funnel plots of comparisons between D2 blockers and Placebo

[DROPOUT DUE TO ANY CAUSE 2](#_Toc27750275)

[DROPOUT DUE TO ADVERSE EVENTS 3](#_Toc27750276)

## DROPOUT DUE TO ANY CAUSE
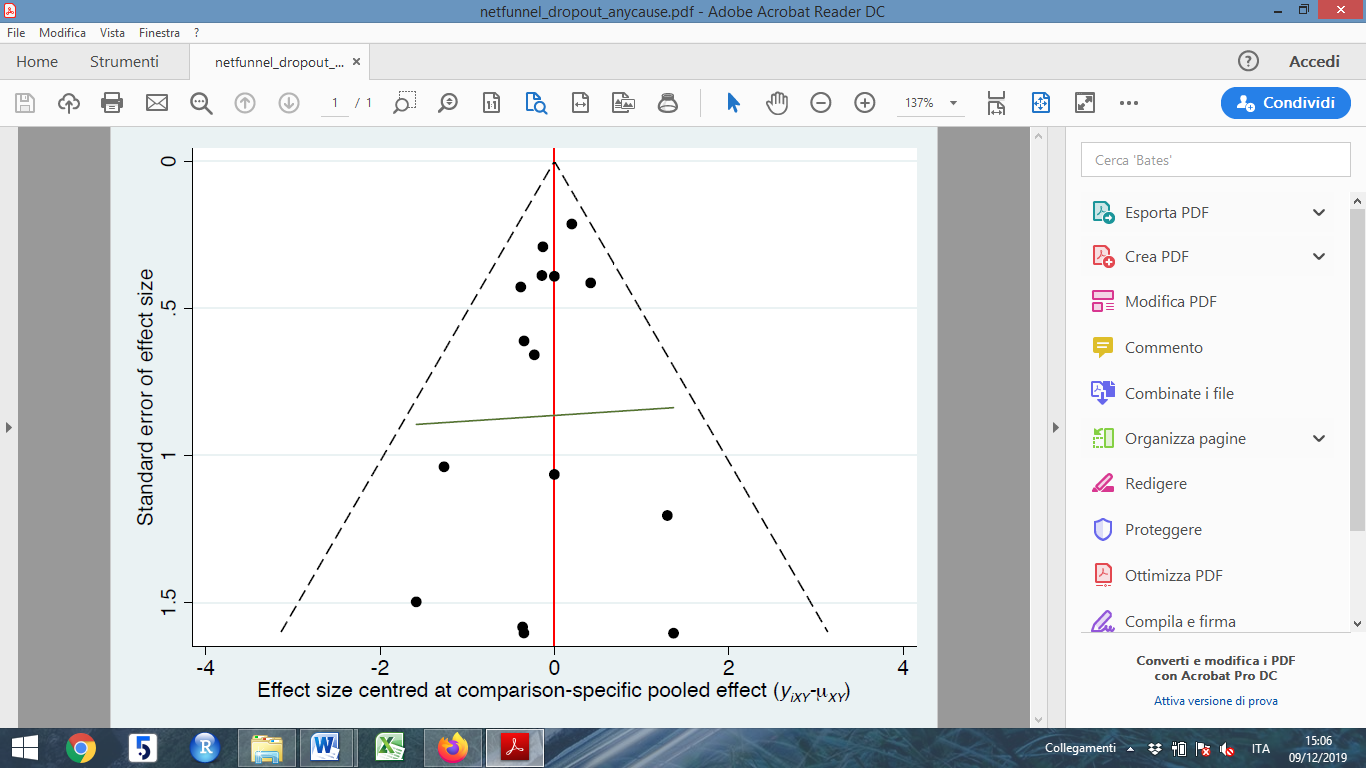


## DROPOUT DUE TO ADVERSE EVENTS


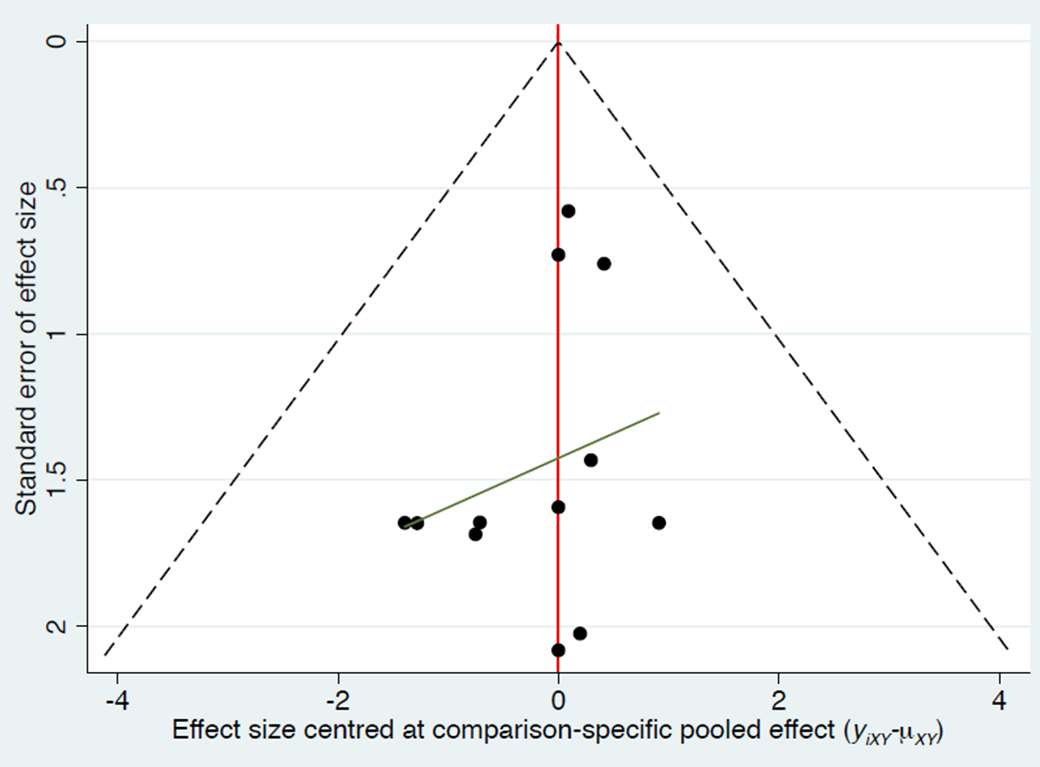

Supplement: Supplementary file 7 — Additional file 7. Funnel plots. [file 12888_2020_2956_MOESM7_ESM.docx]
